# Metabolic characterization of a CHO cell size increase phase in fed-batch cultures

**DOI:** 10.1007/s00253-017-8531-y

**Published:** 2017-09-26

**Authors:** Xiao Pan, Ciska Dalm, René H. Wijffels, Dirk E. Martens

**Affiliations:** 1 Bioprocess Engineering, Wageningen University, PO Box 16, 6700 AA Wageningen, the Netherlands; 2 Synthon Biopharmaceuticals BV, Upstream Process Development, PO Box 7071, 6503 GN Nijmegen, the Netherlands; 3 Faculty of Biosciences and Aquaculture, Nord University, N-8049 Bodø, Norway

**Keywords:** Chinese hamster ovary (CHO) cell, Fed-batch, Antibody production, Phase transition, Metabolic flux analysis, Cell size increase

## Abstract

Normally, the growth profile of a CHO cell fed-batch process can be divided into two main phases based on changes in cell concentration, being an exponential growth phase and a stationary (non-growth) phase. In this study, an additional phase is observed during which the cell division comes to a halt but the cell growth continues in the form of an increase in cell size. The cell size increase (SI) phase occurs between the exponential proliferation phase (also called the number increase or NI phase) and the stationary phase. During the SI phase, the average volume and dry weight per cell increase threefold linearly with time. The average mAb specific productivity per cell increases linearly with the cell volume and therefore is on average two times higher in the SI phase than in the NI phase. The specific essential amino acids consumption rates per cell remain fairly constant between the NI and the SI phase, which agrees with the similar biomass production rate per cell between these two phases. Accumulation of fatty acids and formation of lipid droplets in the cells are observed during the SI phase, indicating that the fatty acids synthesis rate exceeds the demand for the synthesis of membrane lipids. A metabolic comparison between NI and SI phase shows that the cells with a larger size produce more mAb per unit of O_2_ and nutrient consumed, which can be used for further process optimization.

**Electronic supplementary material:**

The online version of this article (10.1007/s00253-017-8531-y) contains supplementary material, which is available to authorized users.

## Introduction

Chinese Hamster Ovary (CHO) cells are the predominant host for the production of therapeutic monoclonal antibodies (mAbs). At the moment, fed-batch is the most common industrial process for CHO cell cultures, due to its ease of operation and flexibility. Three different culture phases can normally be discerned in a fed-batch culture: an exponential growth phase, a stationary phase, and a death phase (Ahn and Antoniewicz 2011; Sengupta et al. 2011; Carinhas et al. 2013). The transitions between these phases are caused by the changes in culture conditions (e.g., nutrient depletion and/or waste accumulation). The transition from the growth to the stationary phase determines the maximum viable cell density (VCD), whereas the transition from stationary to death phase determines the culture longevity. These collectively determine the integral viable cell density (IVCD) which is positively correlated with the final mAb product titer. A better understanding of the underlying mechanisms causing these transitions can be used to develop cultivation processes in which cells grow to higher densities or to remain viable for a longer period of time, which both will result in an increased mAb volumetric productivity. Many studies have compared the metabolic and physiological states of different CHO cell culture phases. The growth phase is characterized by higher specific rates of glycolysis and glutaminolysis and, as a result, also by a higher lactate production rate. During the stationary phase, the metabolism shifts towards a more efficient use of substrates, represented by a lower metabolic flux through glycolysis, a higher flux through the TCA cycle, and often the consumption of lactate. Martínez et al. (2013) showed that a CHO cell line grown in a batch culture had a six times higher energy efficiency when consuming lactate as compared to the situation where lactate was produced. Using isotopic labeling in combination with metabolic flux analysis, Sengupta et al. (2011) and Templeton et al. (2013) both reported higher metabolic fluxes through the pentose phosphate pathway (PPP) and TCA cycle during the stationary phase as compared to the growth phase of CHO cell cultures. Furthermore, Templeton et al. (2013) showed that the high PPP and TCA activities are associated with peak mAb production. Results from Wahrheit et al. (2014) suggested that the metabolic shift in different culture phases of CHO cells is controlled by glycolytic regulation which affects the intracellular pyruvate availability. Ahn and Antoniewicz (2013) reported a higher fatty acids biosynthesis rate than needed by the cells during the stationary phase of CHO cell cultures. Despite that these studies have contributed to the fundamental understanding of CHO cell metabolism and resulted in improved process performances, the mechanisms underlying the phase transition are not yet fully understood, due to the complexity of media and feeds used and the highly dynamic nature of the current fed-batch processes.

In a previous study on the selection of basal media and feeds for CHO cell fed-batch cultures (Pan et al. 2017), it was observed that a cell size increase occurred after the exponential growth phase when ActiCHO feed A/B (GE Healthcare) was used but not when Efficient feed A, B, and C (Gibco™) were used. The cell size increase correlated with higher final product titers. The physiological state of the larger cells and the cause of the size increase were not clear. The aim of this paper is to characterize the metabolic changes that occur upon the cell size increase in order to obtain more insight in the underlying mechanisms that cause the cell size increase and identify possible implications for process design. The metabolic changes are characterized in terms of biomass composition, specific consumption and production rates, and overall metabolic flux distributions using flux balance analysis (FBA).

## Materials and methods

### Cell line and pre-culture

A suspension CHO^BC^® cell clone (BC-P, provided by Bioceros Holding BV, Utrecht, NL) producing a recombinant immunoglobulin G1 (IgG1) was used in this study. Cells were thawed from a working cell bank and maintained in ActiCHO-P medium (GE Healthcare, France) supplemented with 4 mM glutamine (Gibco™, Paisley, Scotland, UK) and 0.5% Anti-clumping agent (Gibco™ Paisley, Scotland, UK). 200 μg/mL Zeocin™ and 5 μg/mL Blasticidin (both from Gibco™, Grand Island, NY, USA) as selection reagents were added during the pre-cultures. Cells were maintained in a 125 mL un-baffled shake flask (Corning, NY, USA) with a 25 mL working volume in a CO_2_ (8%) and temperature (37 °C) controlled incubator. The maintained cultures were sub-cultured every 3 days to 2 × 10^5^ viable cells/mL. The seed train was scaled up through 2 and 20 L rocking bag systems. During the seed train scale-up, the medium described above was used but without the two selection reagents.

### Fed-batch process

Triplicate fed-batch cultures were conducted in 10 L bioreactors (Sartorius Stedim Biotech, France) controlled by BIOSTAT® B-DCU II. Each bioreactor was inoculated with a starting density of 3 × 10^5^ viable cells/mL at a starting volume of 5 L. Culture temperature was controlled at 37 °C, dissolved oxygen (DO) was controlled at 40% by enriched O_2_ flow, pH was controlled at 7.2 by using base and CO_2_. From day 3 onward, feeds were added to each bioreactor daily. First, the glucose concentration was measured. If the concentration was lower than 18 mM, a 45% (*w*/w) glucose solution was added as one bolus to reach a glucose concentration of 28 mM in the reactor. Next, 4.5% (*v*/*v*) ActiCHO feed A, also containing about 500 mM glucose amongst other nutrients, and 0.45% (*v*/*v*) ActiCHO feed B (both from GE Healthcare, USA) per culture volume per day were fed to each reactor. Simethicone antifoam solution was added to each bioreactor prior to inoculation and during the culture when needed.

### Sampling and analysis

For each bioreactor, a 15-mL sample was taken daily before and after the feed addition. Total cell density, viable cell density, and cell diameter were measured using a CedexHiRes® analyzer (Roche, Switzerland). Off-line pH, pCO_2_, glucose concentration, lactate concentration, ammonium concentration and osmolality were measured by a Nova FLEX analyzer (Nova Biomedical, USA). The remaining sample was spun down at 3345×*g* for 15 min and stored at −20 °C for later analysis. On culture day 4, 7, and 10, biomass samples were taken from each bioreactor containing 300 million cells per sample. The samples were spun down at 500×*g* for 10 min and re-suspended in PBS solution (Lonza, Switzerland). Next, the viable cell density was measured again, and each sample was aliquoted into six 15-mL centrifuge tubes with each tube containing 50 million viable cells. The tubes were spun down at 500×*g* for 10 min again after which the PBS supernatant was discarded and the wet cell pellets were stored at −20 °C for later biomass analysis.

Total soluble cellular protein was determined using Lowry Bio-Rad *DC* Protein assay kit (Bio-Rad, NL). Bovine serum albumin (BSA, Sigma-Aldrich) was used as a reference standard. The extraction, separation, and quantification of triacylglyceride (TAG) and polar lipids were performed as described by Breuer et al. (2013) using the sample preparation method 2. Lipid droplets in CHO cells were stained with BODIPY 505/515 (Invitrogen Molecular Probes, Carlsbad, CA) and visualized using a confocal laser scanning microscope (LSM510; Carl Zeiss, Jena, Germany), as described by Cooper et al. (2010). Total cellular carbohydrate content was measured according to the DuBois’ method (DuBois et al. 1956). A glucose solution (Sigma-Aldrich) was used as a reference standard. Cell dry weight (DW) was calculated based on the difference in weight of the tube with the 50 million freeze-dried cells and the pre-weighed centrifuge tube itself.

Compositions of the spent medium including extracellular amino acids, sugars, and organic acids were quantified using NMR (Spinnovation Biologics BV, Oss, NL). IgG1 titer was quantified by Protein-A Chromatography (Agilent, 5069–3639). The N-glycans were quantified by Hydrophilic Interaction Chromatography (HILIC UPLC). A dextran calibration ladder standard (Waters) solution was used to identify the glucose unit of the measured N-glycans. Both mAb quantification and N-glycan analysis were developed by Bioprocess engineering group of Wageningen University.

### Average specific metabolic rates

The average specific metabolic rates were calculated for the NI and the SI phase, respectively. Day 0 and 1 were not considered in calculating the average specific rates for the NI phase, due to a metabolic adaptation period just after inoculation. The average specific production rate of antibody was calculated by averaging the daily specific rates during both the NI and the SI phases. The following equation is used to calculate the specific production rate of a compound x, as described in Pan et al. (2017):

1 $$ {M}_x(t)-{M}_x(0)-{V}_f\times {C}_f={q}_x\bullet {\int}_0^t{X}_{VC} dt $$

where M_x_ (mg; mmol) is the total amount of compound x in a culture, X_VC_ is the number of viable cells in the reactor, V_f_ (mm^3^) is the total volume of feed added, C_f_ (mM) is the concentration of compound x in the feed, and q_x_ (mg·cell^−1^·day^−1^; mmol·cell^−1^·day^−1^) is the cell-specific production rate of the compound x. When the rates are calculated based on cell volume, X_VC_ (mm^3^) presents the volume of viable cells in a culture and q_x_ (mg·mm^−3^·day^−1^ or mmol·mm^−3^·day^−1^) is the cell volume-specific production rate of compound x. The average specific production rates (q) of glucose, amino acids, and organic acids were obtained from a plot of the total amount of production/consumption against the total integral viable cell number (or total integral viable cell volume) using linear regression. Positive values indicate production while negative values indicate consumption. The specific cell growth rate (μ_NI_; day^−1^) during the NI (exponential growth) phase is given by:

2 $$ {\upmu}_{NI}=\raisebox{1ex}{$\mathit{\ln}\frac{X_{VC}(t)}{X_{VC}(0)}$}\!\left/ \!\raisebox{-1ex}{$t$}\right. $$

where X_VC_ is the total number of viable cells in the reactor. During the SI phase in which the cell concentration is constant and the cell volume increases linearly with time, the cell-specific production rate of biomass (q_SI_; day^−1^) is calculated by:

3 $$ {\mathrm{q}}_{SI}=\raisebox{1ex}{$\frac{M(te)-M(ts)}{te- ts}$}\!\left/ \!\raisebox{-1ex}{${X}_{VC}$}\right. $$

where M(te) and M(ts) are the amounts of biomass in a reactor at the end (te) and the start (ts) of the SI phase. In principle, the unit of M is gram dry weight. However, to compare the growth in the SI phase to the exponential growth in the NI phase, the amount of biomass is still expressed in cell number by dividing the dry weight by the dry weight of a cell in the NI phase. In this way, the specific biomass production rate in the SI phase can still be compared to the specific growth rate in the NI phase. The specific growth rate in the NI phase and the specific biomass production rate in the SI phase are used as input values for the subsequent flux balance analysis.

### Flux balance analysis (FBA) and flux variability analysis (FVA)

A basic model of CHO cell primary metabolism was developed starting from an earlier published basic model for mammalian cells (Martens and Tramper 2010). The reactions in the model were cross-checked with the iCHOv1 model (Hefzi et al. 2016). Several modifications were made based on the Kyoto Encyclopedia of Genes and Genomes (KEGG) database, the iCHOv1 model, and the measurements done in this study (ESM 1).

A Chi-square test was used to check whether the nitrogen balances closed for both phases. The FBA was conducted using the constraint-based reconstruction and analysis (COBRA 2.0) Toolbox (Schellenberger et al. 2007), using MATLAB software (The MathWorks, Inc.). Geometric FBA (Smallbone and Simeonidis 2009) was performed to find a unique solution for the underdetermined parts of the metabolic model. The growth rate was used as an objective function for the model. The measured cell-specific production rates were used as inputs for FBA. To study the possible range of flux values that results in the same optimal growth rate while still meeting all constraints, flux variability analysis (FVA) was performed using the COBRA Toolbox (ESM 1).

## Results

### Cell growth

**Fig. 1:**
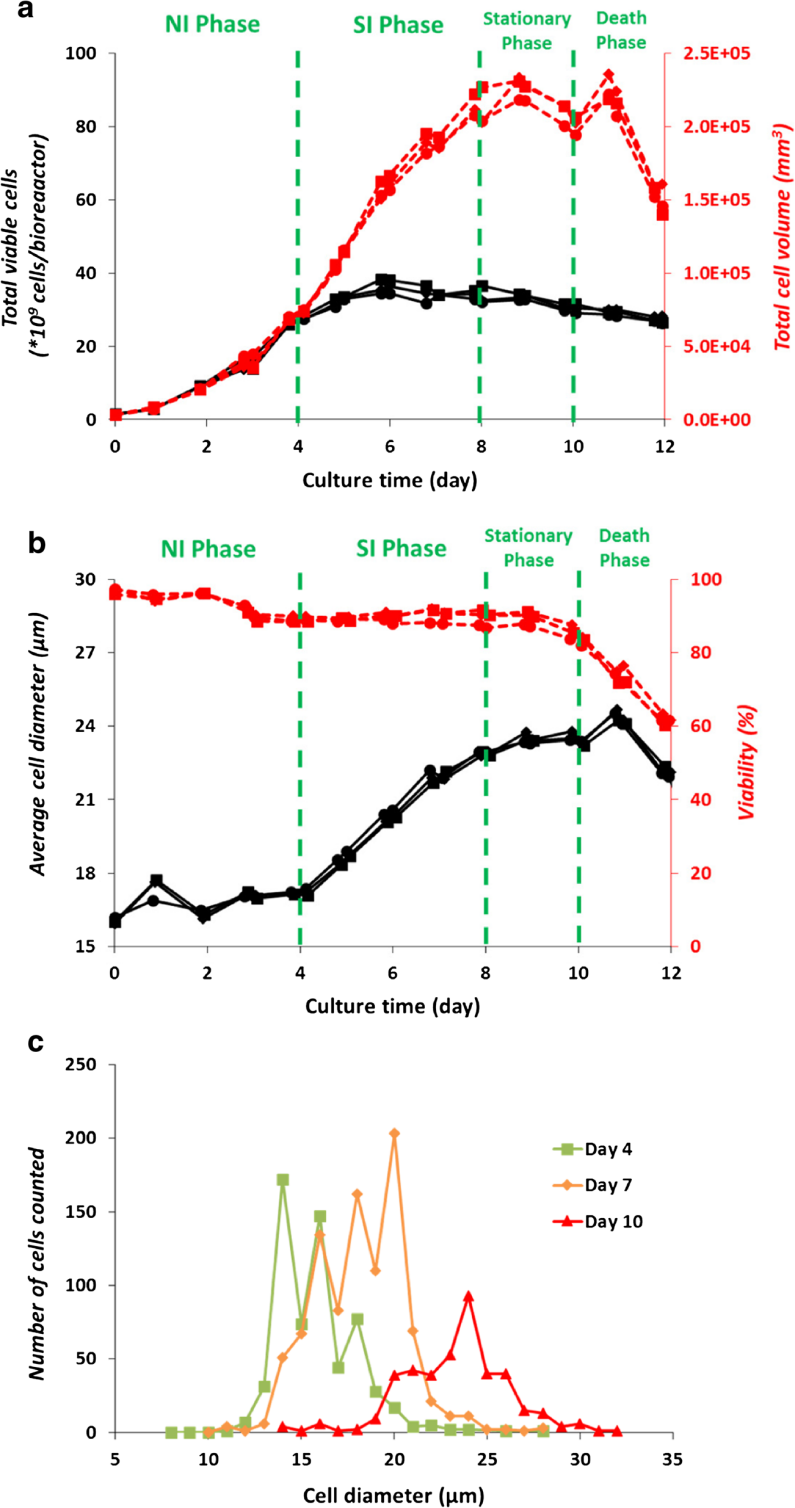
**a** Total number of viable cells (×10^9^, solid lines) and total volume of viable cells (mm^3^, dotted lines), and (**b**) average cell diameter (μm, solid lines) and viability (%, dotted lines) of reactor 1 (closed circles), 2 (closed diamonds), and 3 (closed squares). Cultures were divided into a number increase (NI) phase, a size increase (SI) phase, a stationary phase, and a death phase. (**c**) Cell diameter (μm) distribution during a representative fed-batch culture on day 4 (closed squares), 7 (closed diamonds), and 10 (closed triangles)

Based on cell concentration, the growth curve of CHO cells normally consists of an exponential cell proliferation phase, a stationary phase, and a death phase. In this study, an additional cell size increase phase is reported (Fig. 1), resulting in four phases for this CHO cell fed-batch process being: (i) A phase in which the cell concentration increases exponentially (day 0 to 4) and the volume per cell remains constant (the number increase or NI phase). (ii) A phase in which the cell size increases nearly linear (*R* ^2^ = 0.99) in time (day 4 to 8) and the cell concentration stays approximately constant (the size increase or SI phase). (iii) A short stationary phase (day 8 to 10) in which the biomass concentration remains approximately constant and the culture viability is always higher than 80%. (iv) A death phase (day 10 to 12) in which the viability declines and is always lower than 80%. Since the observed change from growth in cell concentration to growth in cell size has not been reported in regular fed-batch processes and can have consequences for process development, this study mainly focuses on the NI phase and the SI phase.

### Metabolites profiles

**Fig. 2:**
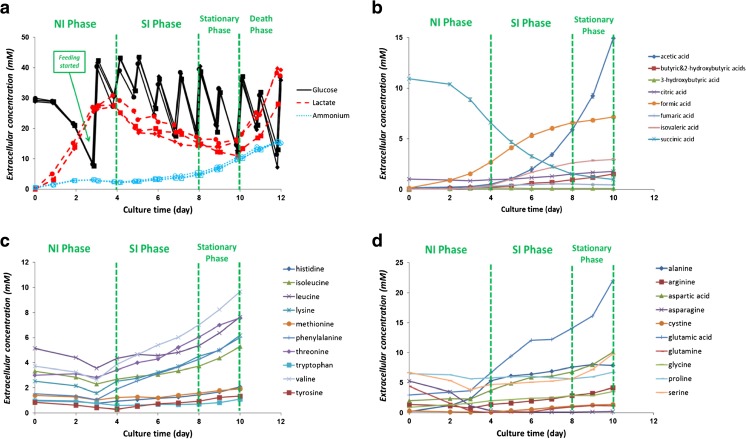
**a** Extracellular glucose (solid lines), lactate (dotted lines with closed marks), and ammonium (dotted lines with open marks) concentrations (mM) of reactor 1 (circles), 2 (diamonds), and 3 (squares). (**b**) Extracellular concentrations of measured organic acids. (**c**) Extracellular concentrations of essential amino acids. (**d**) Extracellular concentrations of non-essential amino acids. Feeding started on day 3. For glucose, lactate, and ammonium, samples were taken daily before and after feed addition. For the other organic acids and amino acids, samples were taken daily before feed addition. Error bars for all graphs indicate the standard deviation for the triplicate bioreactors

To identify possible limiting or inhibitory compounds and to characterize metabolism in the different phases, extracellular concentrations of substrates and products were measured. Concentrations of extracellular glucose, lactate, and ammonium are shown in Fig. 2a. The 45% glucose solution is added on culture day 3, 7, 8, and 10 when the glucose concentration is lower than 18 mM, as described in materials and methods. Next to this, the ActiCHO feed A&B are added daily. On the days other than 3, 7, 8, and 10, the increases in culture glucose concentrations were caused by the addition of ActiCHO feed A only. Using this feeding strategy, the glucose concentration in each bioreactor stayed above 5 mM throughout the fed-batch cultures. Furthermore, since the ActiCHO feed A also contains glucose, the culture glucose concentration after feeding can be higher than 28 mM. Lactate is produced during the NI phase (day 0–4) until nearly 30 mM and is consumed at a lower rate during the SI phase and stationary phase (day 4–10), followed by production again during the death phase (day 10–12). Ammonium concentrations stay constant at values lower than 5 mM until culture day 7 and start to increase gradually thereafter up to 15 mM on day 12. Production of formic acid (up to 7 mM at day 10) and acetic acid (up to 15 mM at day 10) are observed in the cultures (Fig. 2b). Succinic acid is present in the basal medium and is consumed during the culture (Fig. 2b). None of the essential amino acids is depleted during the fed-batch cultures (Fig. 2c). The gradual increase in their concentrations after day 3 is caused by the feed addition, since these essential amino acids cannot be made by CHO cells from the other compounds. Note that for some of the non-essential amino acids the cell may not be able to synthesize them fast enough to support growth making them essential for a robust process, consequently, they also need to be present in the medium and feed. For some of the non-essential amino acids, asparagine, glutamine, and cyst(e)ine (cysteine is measured in the form of cystine in this study) are consumed during the NI phase and are depleted around day 4 (Fig. 2d). Glutamine, which is not present in the feed, is produced after day 7. Cyst(e)ine concentration increases after day 5. The complete set of raw data for the extracellular compounds and the composition of both feeds can be found in ESM 2.

### Cell-specific rates vs. volume-specific rates

In order to obtain insight into the relationship between cell volume and the specific consumption and production rates of nutrients and products, including mAb, their metabolic rates were calculated and compared in this section. The rates are calculated on a per cell and per cellular volume basis for the NI and the SI phase, respectively.

**Fig. 3:**
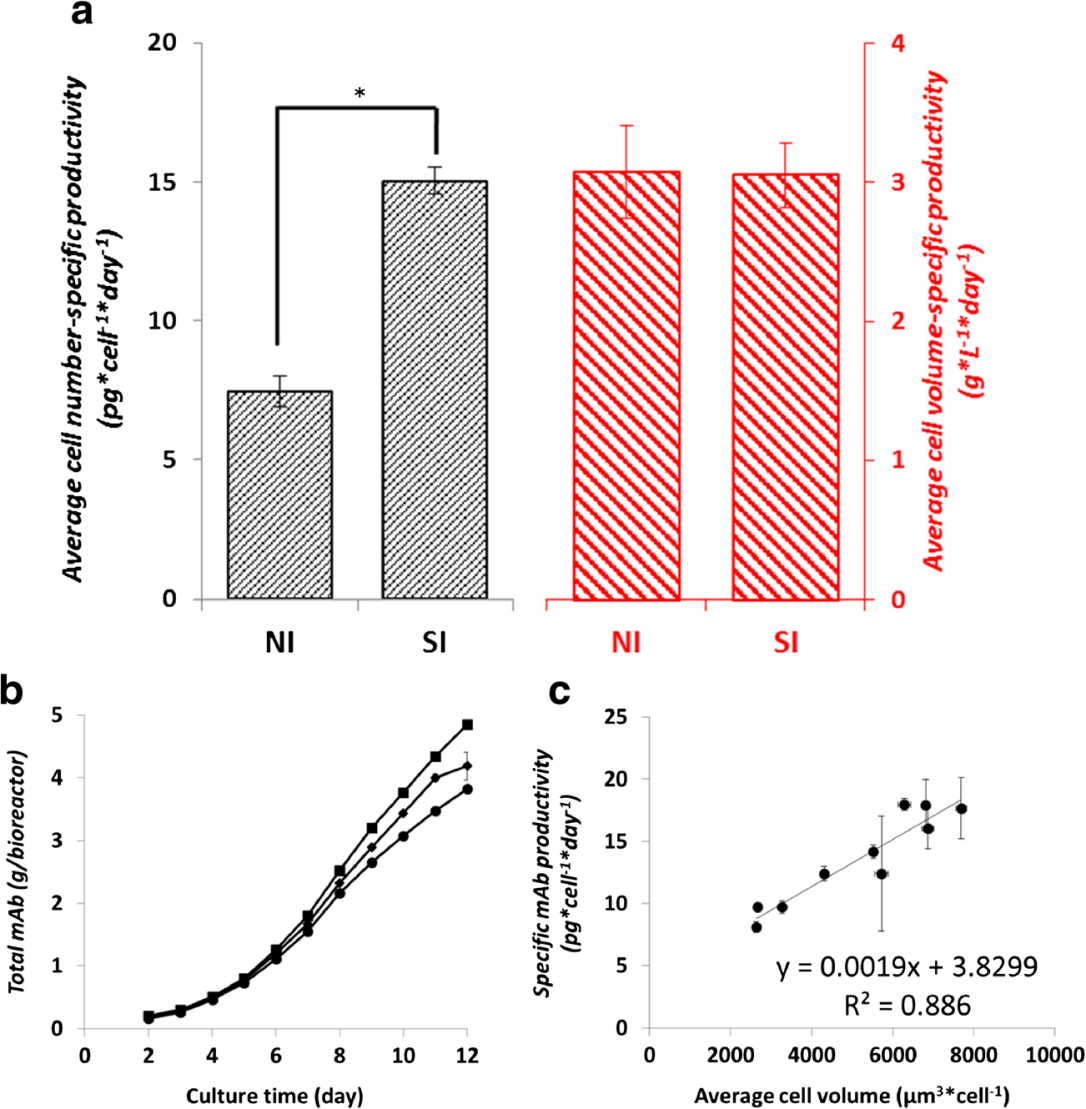
**a** Average cell number-specific (pg × cell^−1^ × day^−1^) and average cell volume-specific (g × L^−1^ × day^−1^) mAb productivity compared between the number increase (NI) and size increase (SI) phase of the cultures. *: significantly different (*P* < 0.05). **b** Total mAb produced (g) in reactor 1 (closed circles), 2 (closed diamonds), and 3 (closed squares). **c** Plot of specific mAb productivity (pg × cell^−1^ × day^−1^) against cell volume (μm^3^). Calculation was done per day from day 2 through day 12. Error bars of graph (a) and (c) show the standard deviation of triplicate fed-batch cultures. Error bars of graph (b) show the standard deviation of triplicate measurements for each fed-batch culture

**Fig. 4:**
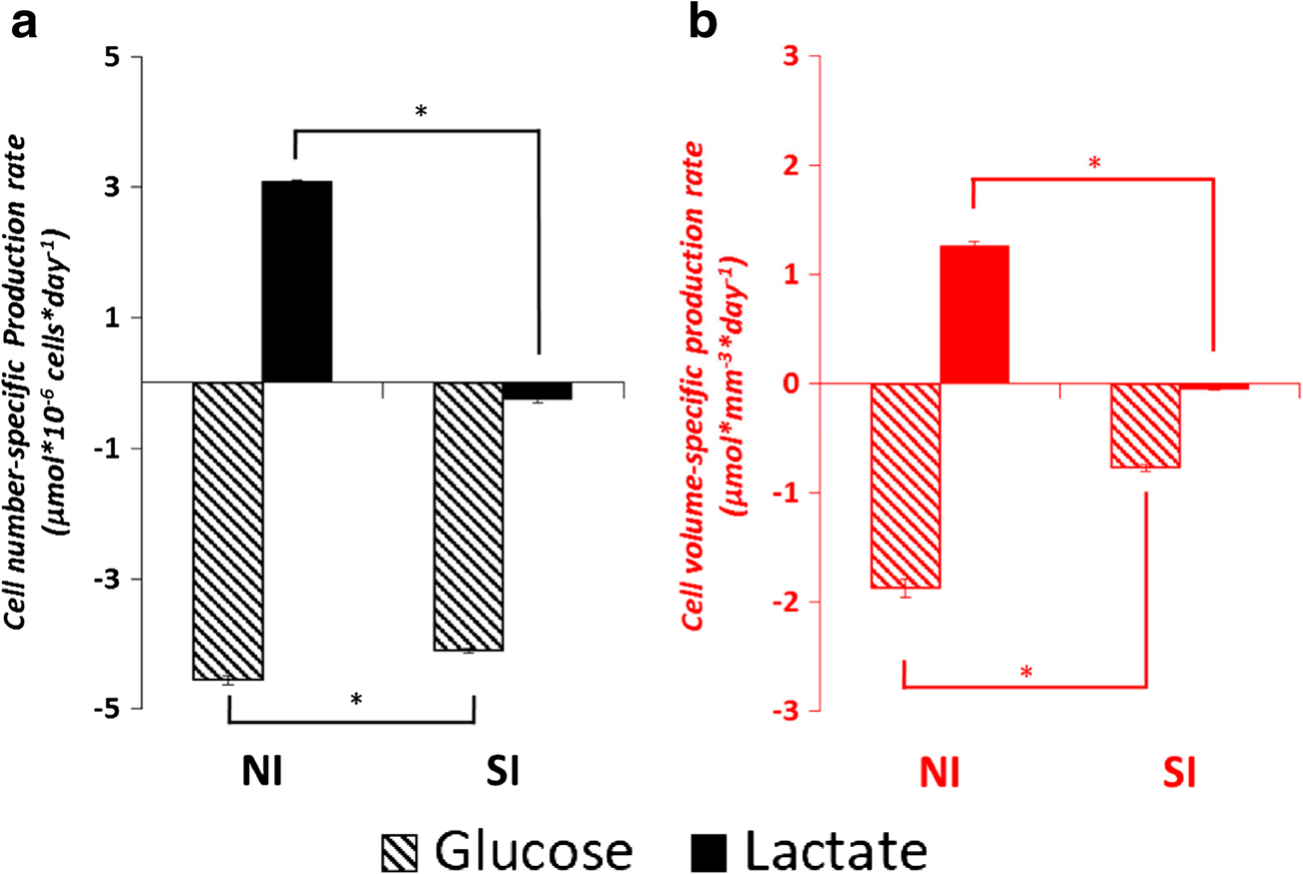
(a) Cell number-based specific rates (μmol × 10^−6^ cells × day^−1^) and (b) cell volume-based specific rates (μmol × mm^−3^ × day^−1^) of glucose consumption and lactate production/consumption during the number increase (NI) and size increase (SI) phase of the fed-batch cultures. Positive values indicate production and negative values indicate consumption. Error bars show the standard deviation of triplicate fed-batch cultures. *: significantly different (*P* < 0.05)

**Fig. 5:**
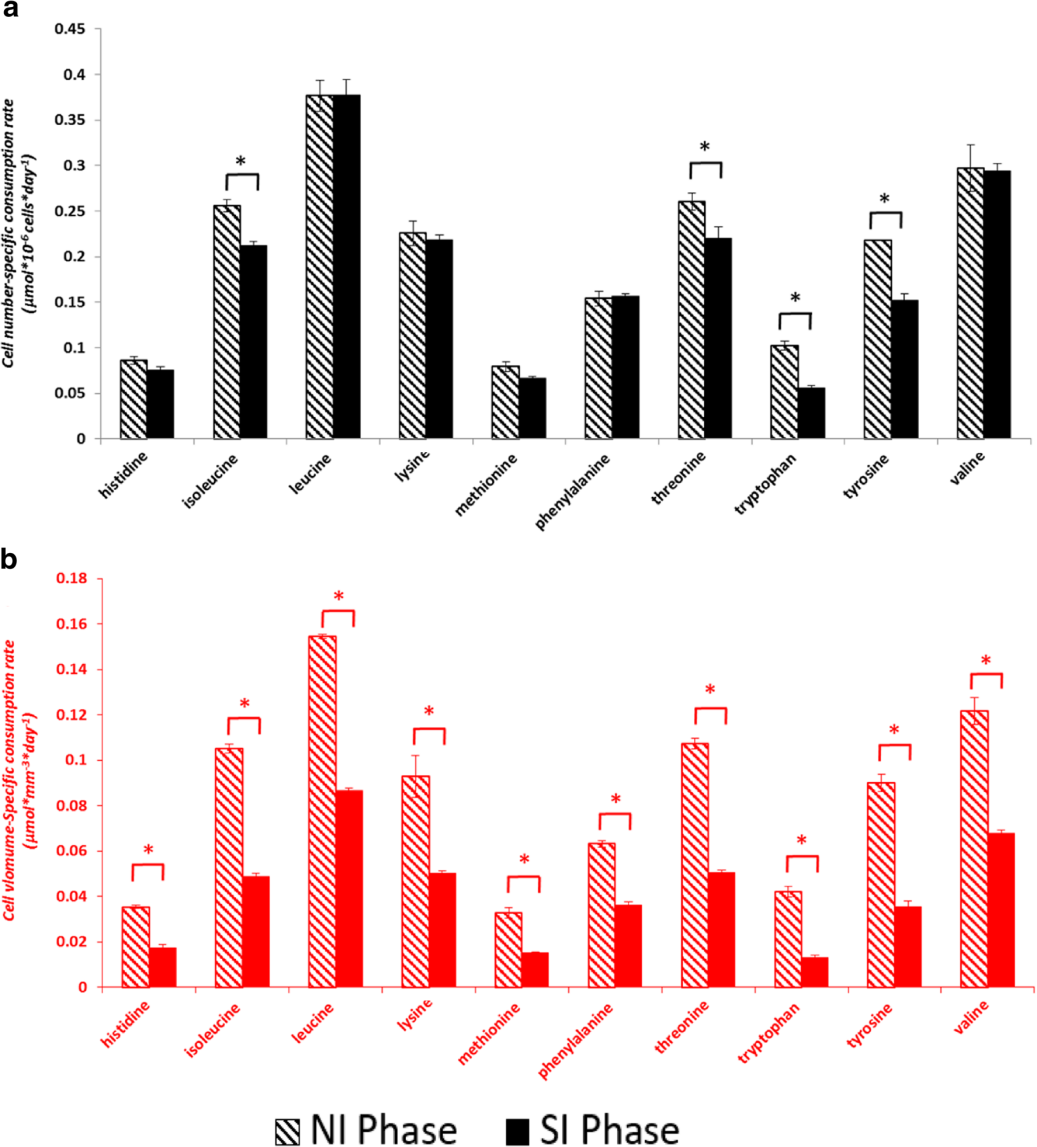
**a** Cell number-specific consumption rates (μmol × 10^−6^ cells × day^−1^) and (**b**) Cell volume-specific consumption rates (μmol × mm^−3^ × day^−1^) of essential amino acids during the number increase (NI) and size increase (SI) phase of the fed-batch cultures. Error bars show the standard deviation of triplicate fed-batch cultures. *: significantly different (*P* < 0.05)

The average specific mAb productivities calculated based on cell-volume and cell-number are shown in Fig. 3a. Average specific productivity expressed per cell is twofold higher (15 compared to 7.5 pg × cell^−1^ × day^−1^) in the SI phase compared to the NI phase. When calculated per cell volume, the specific productivities in the two phases are not significantly different, indicating that the mAb productivity of a cell is related to its volume. This becomes clearer when the productivity per cell is plotted against the cellular volume, which results in a linear correlation between average cell volume and specific mAb productivity as shown in Fig. 3c. The specific glucose consumption based on cell number shows only a slight decrease (4.5 to 4.1 μmol × 10^−6^ cells × day^−1^) from the NI to the SI phase. The decrease is larger (1.9 to 0.8 μmol × mm^−3^ × day^−1^) when the rate is calculated based on cell volume. Thus, the specific glucose consumption rate is related more to the cell number and less to the cell volume. Lactate is produced during the NI phase at a high rate (3 μmol × 10^−6^ cells × day^−1^; 1.3 μmol × mm^−3^ × day^−1^) and is consumed during the SI phase at a much lower rate (0.25 μmol × 10^−6^ cells × day^−1^; 0.05 μmol × mm^−3^ × day^−1^) (Fig. 4). The cell number-specific and cell volume-specific consumption rates of ten essential amino acids during the NI and the SI phase are shown in Fig. 5. When the consumption rates are expressed based on cell number, six out of ten essential amino acids do not show a significant difference between the two phases. However, as the cell volume increases in the SI phase, the cell volume-specific consumption rates of all the essential amino acids show significant drops. This implies that the consumption of the essential amino acids is more related to the cell number than to the cell volume.

### Biomass composition

**Fig. 6:**
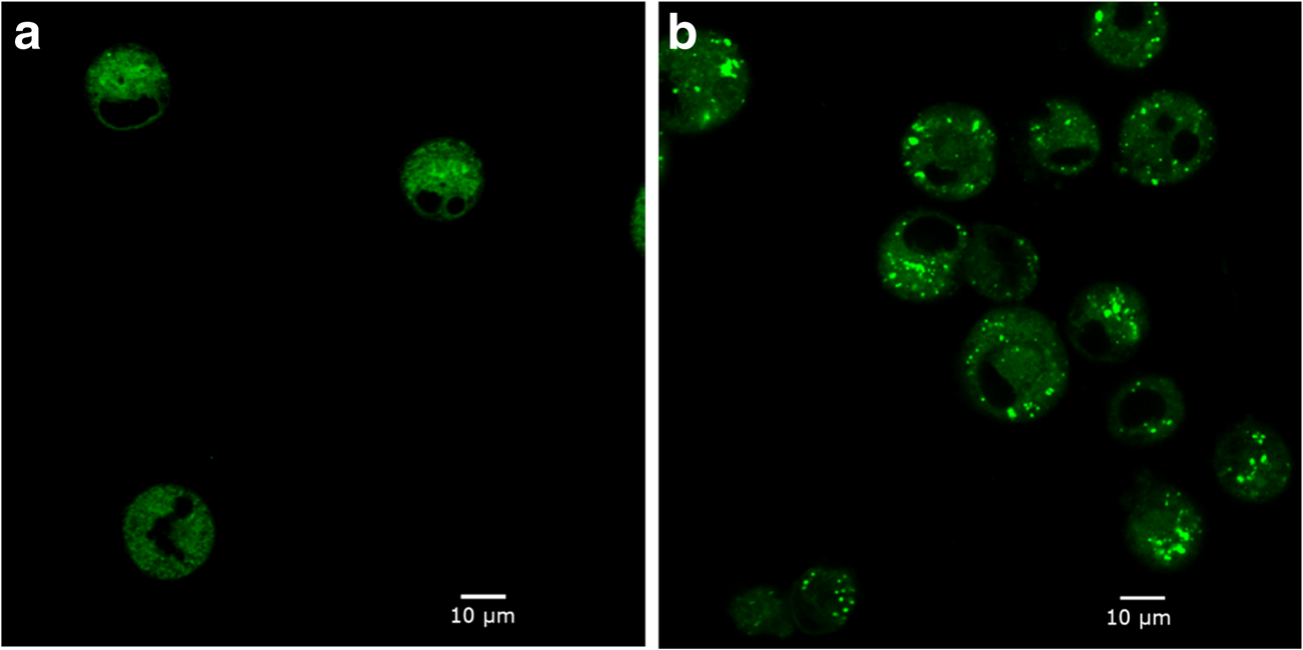
Lipid droplets stained with BODIPY 505/515 (green) during the CHO cell fed-batch on culture day 3 (**a**) and culture day 6 (**b**)

The composition of biomass with respect to protein, fatty acids, carbohydrate, RNA, and DNA is shown in Table 1 as a percentage of the cell dry weight (DW). The amino acid composition of the cellular protein and the fatty acids composition of the lipids stayed constant during the fed-batch culture (the average values are given in ESM 3 Table S1 and S2). The cell DW increased linearly with the cell volume over the culture time, from 770 pg/cell on day 4 to 2440 pg/cell on day 10 (Table 1). Furthermore, the relative composition of the biomass shifted during the cultures. The protein content in the biomass decreased from 74.5 to 67% whereas the fatty acid content increased from 7.2 to 10.3% from culture day 4 until culture day 10. A microscopic image (Fig. 6) shows a clear accumulation of lipid droplets during the SI phase. These droplets are not composed of triacylglyceride (TAG) (data not shown). The sum of all biomass components adds up to around 90% of the cell DW.

**Table 1:** CHO cell biomass composition during the fed-batch cultures

Biomass composition	Day 4	Day 7	Day 10	Hybridoma (Xie and Wang 1994)	Hybridoma (Bonarius et al. 1996)
Protein (%)	74.5 ± 1.0	70.0 ± 1.2	67.0 ± 1.0	72.9	70.6
Fatty acids (%)	7.2 ± 0.2	8.4 ± 0.3	10.3 ± 0.1	13.5 ^b^	9.7 ^b^
Carbohydrate (%)	4.0 ± 0.1	3.8 ± 0.2	4.1 ± 0.1	3.5	7.1
RNA ^a^ (%)	6.0	5.6	5.4	3.8	5.8
DNA ^a^ (%)	1.6	0.8	0.6	1.4	1.4
Total (%)	93.1 ± 1.4	88.6 ± 1.7	87.4 ± 1.2	95.1	94.6
Cell dry weight (pg/cell)	770 ± 47	1530 ± 77	2440 ± 86	250	470
Cell volume (mm^3^/10^6^ cells)	2.7 ± 0.02	5.5 ± 0.16	6.9 ± 0.14	N/R	1.8

All percentage values are in w/w %

^a^Not measured in this study, data were calculated by assuming a constant DNA content per cell and a constant ratio of RNA to protein

^b^Values for lipid including glycerol

± Standard deviation for biological triplicates

N/R not reported

### Flux balance analysis

**Fig. 7:**
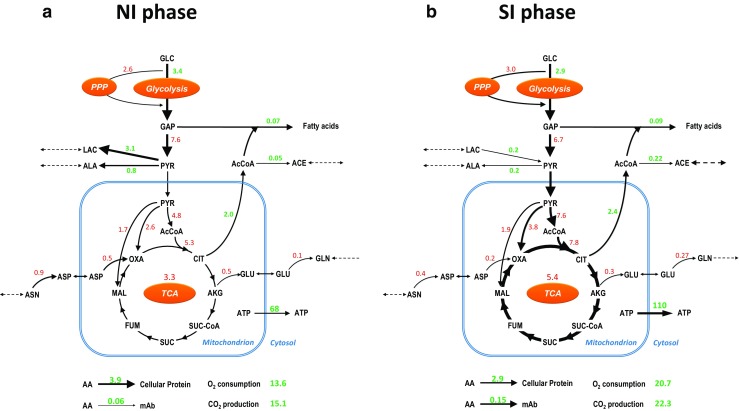
Major metabolic flux changes between (**a**) the number increase (NI) and size increase (SI) phase during the fed-batch cultures. The line thickness indicates the relative flux changes between the two presented phases. The average flux of the TCA cycle is represented by the average fluxes between the flux from citrate (CIT) to α-ketoglutarate (AKG) and the flux from succinate (SUC) to fumarate (FUM). The colors of the flux values indicate the flux variability result between the two phases. The green values show no overlap of the variable flux values between the two phases whereas the red values show there is overlap. The unit for flux values is mmol × 10^−9^ cells × day^−1^

Geometric flux balance analysis (FBA) is carried out to further investigate the shift of the overall metabolic flux distribution from the NI phase to the SI phase. The FBA results for both phases are summarized in Fig. 7. The metabolic fluxes are expressed per cell because the uptake rates of essential amino acids and glucose and the biomass formation rate are constant on a per cell basis in the NI and SI phase. On a per volume basis rates are continuously decreasing during the SI phase due to the increase in cell volume. During the SI phase, the change in biomass content per cell and in biomass composition is considered in the biomass equation in the FBA model. The flux variability analysis (FVA) shows that the flux variability results in overlapping flux values with respect to the two different phases for the PPP as well as for the TCA cycle, as indicated by the red numbers in Fig. 7. This means the value of these fluxes cannot be identified as being different between the two phases, which is further addressed in the discussion. During the NI phase, high fluxes through lactate and alanine production are calculated. During the SI phase, lactate metabolic flux shifts to a consumption and the flux from pyruvate to alanine decreases. Higher O_2_ consumption and CO_2_ production fluxes (~1.5-fold higher) are calculated for the SI phase, which agrees with the higher ATP generation flux in the mitochondria. The ATP is transported to the cytosol through ATP/ADP translocases and used for biomass and product synthesis, and for cell maintenance. The cellular protein formation rate decreases by 25% whereas the mAb formation increases by 250% during the SI phase. In the NI phase, mAb production is about 1.5% of the total protein synthesized, whereas in the SI phase this becomes 5%. In the SI phase, the fatty acids synthesis flux is slightly higher and the flux from AcCoA to acetic acid is also higher than in the NI phase. For the complete result of FBA and FVA, see ESM 1.

### mAb N-glycan composition

**Fig. 8:**
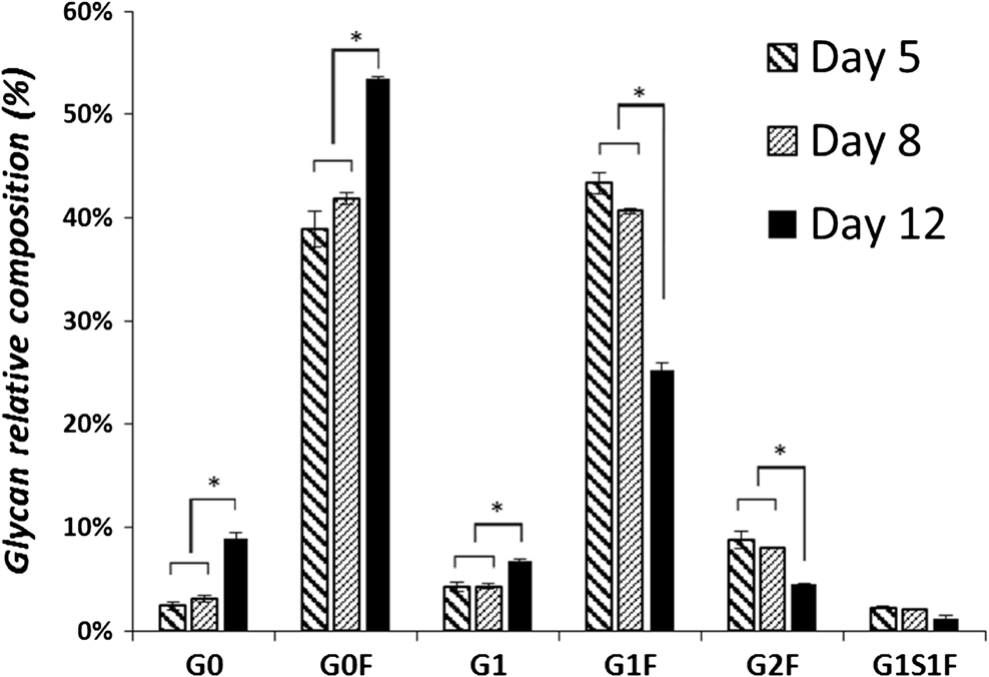
Relative mAb N-glycan composition (%) of the 10 L cultures. N-glycans with different numbers of terminal residuals (G: galactose, F: fucose, S: sialic acid) are shown. Error bars show the standard deviation of the triplicate runs. *: significantly different (*P* < 0.05)

The glycosylation profile of a mAb is routinely analyzed throughout process development to ensure process consistency. In order to investigate whether or not the mAb glycosylation changes as cell size increases, the N-glycan composition of the produced mAb is measured on culture day 5, 8 and 12 (Fig. 8). The relative composition of N-glycans does not show differences between culture day 5 and 8, indicating that the mAb glycosylation does not change as cell size increases. Differences on day 12 are observed which is most likely related to cell death and release of degradative enzymes, since the culture viability drops to 60% on day 12.

## Discussion

In this study using ActiCHO medium as basal medium and Actifeed A and B as feeds, a cell size increase phase was observed during which the cell concentration remained approximately constant, while the cell volume increased almost three times. A previous study (Pan et al. 2017) showed that the cell size increase in CHO fed-batch processes is clone and medium/feed dependent. Furthermore, an experiment with the same process and medium system but with a lower feed addition rate, the same extent of cell size increase was observed (data not shown), implying that the cell size increase is not merely caused by nutrient oversupply. However, several non-essential amino acids were depleted around day 4 (Fig. 2d), which might play a role in the metabolic transition from the NI to the SI phase.

The cell diameter distribution during the fed-batch cultures (Fig. 1c) showed that the increase in cell diameter occurred for the whole cell population instead of only a sub-population. It was also established in this study that the cell size increase is due to a proportional increase in biomass content (Table 1), rather than due to a cell swelling effect through the uptake of water and salts. The cell DW measurement resulted in higher values (770 pg/cell or higher) than commonly reported for CHO cells (300–400 pg/cell) (Altamirano et al. 2001; Niklas et al. 2011; Martínez et al. 2015). This can be explained by the larger cell size (17 μm diameter on day 4) of the CHO cell line used in this study, compared to those reported (12–14 μm diameter) (Martínez et al. 2015). We applied the same DW measurement for another cell line with an average cell diameter of 14 μm at a viability higher than 90%, which resulted in ~330 pg/cell (data not shown). Furthermore, the amino acid composition of the cellular protein and the fatty acids composition of the lipids analyzed in this study (ESM 3 Table S1 and S2) are comparable to those reported for other CHO cells (Berlin et al. 1996; Selvarasu et al. 2012). With respect to the content of protein, lipids, carbohydrates DNA, and RNA, such data could not be found in or derived from CHO cell studies and therefore, a comparison to the biomass composition of hybridoma cells (Xie and Wang 1994; Bonarius et al. 1996) is shown (Table 1). As can be seen, the compositions are more or less comparable.

The results for the CHO cell line used in this study show that the mAb specific productivity is directly proportional to the cell volume (Fig. 3a, c), and further confirm that the mAb glycosylation profile does not change as the cell size increases (Fig. 8). This is in agreement with Lloyd et al. (2000) who argued that the cell size is the major cellular determinant of productivity rather than the cell cycle phase. In addition to this, Edros et al. (2014) and Khoo and Al-Rubeai (2009) showed that the increase in specific productivity is directly related to the increase in cell volume due to increase in transcription, translation, and secretion machinery. Kim et al. (2001) reported that the enhancement of the specific thrombopoietin productivity is linearly correlated with the increase in cell size for nine CHO cell clones generated using the same transfection process. In a number of studies the relation between cell size and specific productivity was used to enhance mAb productivity, by directly or indirectly increasing the cell size, for example, through cell cycle arrest (Carvalhal et al. 2003; Bi et al. 2004), hyperosmotic pressure (Sun et al. 2004; Kiehl et al. 2011), and mild hypothermia (Tait et al. 2013; Martínez et al. 2015).

Acetic acid and formic acid accumulation were observed during the cultures (Fig. 2b and ESM 3 Fig. S1). Possibly the acetic acid is formed from acetyl-CoA by one of the thioesterases that may be present in CHO cells (Bernson 1976). This is further supported by the formation of isovaleric acid during the SI phase (Fig. 2b), which is formed from isovaleric acid-CoA as a breakdown product of leucine (Tanaka et al. 1966) and also requires the activity of an esterase. The formic acid concentration in this experiment reached 4 mM on day 5 (Fig. 2b), which concentration was shown to inhibit CHO cell growth (Ihrig et al. 1995). Formic acid is considered a breakdown product of serine (Carinhas et al. 2013; Duarte et al. 2014). In the present study, formic acid is mainly produced by the breakdown of serine through folate metabolism (ESM 1), which is also reported by Carinhas et al. (2013) and Duarte et al. (2014) and a small fraction is generated in the breakdown of tryptophan and during synthesis of cholesterol (ESM 1). The high breakdown rates for some amino acids are likely caused by the overfeeding of these nutrients after the NI phase as can be seen in Fig. 2. In addition, lactate concentration showed a peak at 30 mM at around the point where the transition from the NI to SI phase occurred (Fig. 2). However, the lactate concentration in this study is not expected to be the trigger of the phase transition, since the cell clone used in this study was observed to be able to grow in cell concentration at a lactate concentration up to 40 mM (data not shown).

During the SI phase, the slightly higher fatty acids synthesis fluxes (Fig. 7) occurred together with the formation of lipid droplets (Fig. 6). Formation of lipid droplets has been reported in CHO cells (Liu et al. 2004). In addition, high fatty acids synthesis rate during stationary phase was previously reported in a CHO cell study using ^13^C isotopic tracers (Ahn and Antoniewicz 2013). However, the fate of the excessive fatty acids was unclear in that study. We hypothesize that the accumulation of lipids in the present study is mainly caused by a maintained fatty acids synthesis rate in combination with a decreased membrane lipid demand during the SI phase. A slight increase in the fatty acid synthesis flux from the NI (0.07) to the SI (0.09) phase (Fig. 7) is observed. The amount of extra NAD(P)H needed for this is negligibly small compared to the total NAD(P)H oxidation flux. The slight increase in the fatty acid synthesis flux, therefore, is not expected to largely affect the overall redox rearrangement and through this play a role in the change in lactate metabolism.

### Metabolic patterns of cell size increase

Based on FVA, higher O_2_ consumption and oxidative phosphorylation fluxes were calculated in the SI phase as compared to the NI phase. This results in a higher ATP synthesis flux during the NI phase, which is reflected in the higher ATP translocation flux from mitochondria to cytosol in the SI phase (Fig. 7). The higher flux through oxidative phosphorylation means that more NADH is generated in the SI phase. The NADH can be generated in glycolysis and the TCA cycle or by the combined activity of the PPP and transhydrogenase systems. However, the fluxes in these pathways cannot be independently calculated (shown by the red flux values in Fig. 7) and thus it remains unknown whether the increased O_2_ consumption and oxidative phosphorylation activity are due to an increase in TCA flux or an increased flux through the PPP or both. Bi et al. (2004) reported a significant increase in mitochondrial activity (1.6-fold) and mitochondrial mass (twofold) for a CHO cell line with a similar cell volume increase (three- to fourfold). Furthermore, Zagari et al. (2013) showed that the lactate metabolism shift from production to consumption was correlated with higher mitochondrial activity. The same lactate metabolic shift also occurred in the SI phase of our study, which also may point to an increase in TCA cycle activity during the SI phase. These studies support that the increased NADH generation flux during the SI phase is mainly generated from a higher flux through the TCA cycle. However, Sengupta et al. (2011) and Templeton et al. (2013) showed by using isotopic labeling that the flux through both the oxidative PPP as well as through the TCA cycle increased during the non-growth phase. In addition, Ahn and Antoniewicz (2011) reported a significant increase in oxidative PPP together with a constant TCA flux from the growth phase to the stationary phase. However, in these last three studies, the increase in cell size was not reported and thus the metabolic condition of the non-growth phase in their studies may not be fully illustrative for the SI phase of this study. In conclusion, increased O_2_ consumption and oxidative phosphorylation fluxes are calculated and it seems most likely that an increased TCA cycle flux is the cause for the faster NADH generation in the SI phase.

The increased NADH demand and likely higher TCA cycle activity to generate this NADH could result in a lower NADH/NAD ratio and a lower pyruvate concentration in the cell. This might be related to the metabolic switch from lactate production to consumption, since the conversion of lactate to pyruvate generates NADH and the pyruvate that is generated can support the higher TCA flux. The higher flux through oxidative phosphorylation may have two causes: (i) a higher energy demand of cells in the SI phase for maintenance and/or growth. This may be directly related to cell size, but may also be related to other factors that change over time like for example the ammonium concentration. The energy requirement for maintenance can increase when cells are exposed to ammonium due to its competitive inward transport over potassium ions via the Na^+^/K^+^-ATPase and the Na^+^K^+^2C1^−^ cotransporter (Martinelle and Häggström 1993). However, the ammonium is not likely to cause an increase in energy demand since its concentration remains reasonably constant between the NI and SI phase. (ii). A decrease in mitochondrial efficiency by uncoupling of the oxidative phosphorylation. An “uncoupler” (i.e., leaky membrane) enables protons to flow back without passing through the ATP synthase, therefore, less ATP is generated from the same amount of NADH and FADH_2_ (i.e., a lower P/O ratio) (Terada 1990). If the demand for ATP stays the same, a higher flux through oxidative phosphorylation and a higher O_2_ consumption rate are needed to meet this demand. The thioesterases responsible for the production of acetic acid and isovaleric acid as mentioned before may also be responsible for the release of free fatty acids that are known to uncouple the oxidative phosphorylation (Pressman and Lardy 1956; Skulachev 1991). The slightly higher fatty acids synthesis fluxes during the SI phase could be related to this.

### Implications of cell size increase during a culture process

The results of this study stress that cell size and the amount of biomass per cell should be taken into account during the process characterization. Based on cell density measurement alone, the threefold increase in biomass observed in this work would not have been captured (Fig. 1), and the cell size increase phase would have been mistakenly identified as part of the stationary phase. In fact, a two-micron increase in cell diameter, for instance, from 14 to 16 μm, may already lead to a 50% increase in total cell volume or biomass. Furthermore, it can skew the process interpretation such as final biomass concentration reached, mAb productivity, and cellular metabolism. Similarly, skewed biological interpretation of a transcriptomic study due to cell size changes was reported by Fomina-Yadlin et al. (2014). The occurrence of cell size increase is also important for feed development. Since biomass continues to grow after the exponential cell concentration increase phase, the nutrients provided to the culture should contain not only nutrients assigned for maintenance and mAb production reactions, but also all the precursors and building blocks for biomass growth. In this specific culture process, a simple feeding concept would be to keep the feeding rate of essential amino acids at a per cell level the same during the SI phase and the NI phase, since the cell number-specific consumption rates remain fairly constant (Fig. 5a).

The present study further shows that during the SI phase the average mAb production per cell increased by a factor 2 (Fig. 3), while the average O_2_ consumption and CO_2_ production increased only by a factor 1.5 (Fig. 7). In addition, compared to the NI phase, during the SI phase a higher proportion of the amino acids taken up from the medium is used for mAb synthesis (Fig. 7). Collectively, the amount of mAb production per mol of oxygen and amino acid consumed increases for larger cells, which can be used for further process optimization. Especially for high-density perfusion processes where O_2_ and/or CO_2_ transfer may be limiting, it may be favorable to have larger cells.

This study demonstrates that cell size is an important factor for characterizing and optimizing a cell culture process. Correlations between the cell size increase and metabolic changes were found. However, further research is needed to understand the relationship between changes in cell size and changes in metabolism. In addition, studies on the cellular pathways that control cell size, such as the mammalian target of rapamycin (mTOR) pathway (Rohde et al. 2001; Fingar et al. 2002), and the extracellular stimuli involved could promote mechanistic understandings for the cell size increase, and enable controlled cell size manipulation during a culture process.

## Electronic supplementary material

ESM 1 (PDF 1196 kb)
