# Supplementary material for: Metabolic characterization of a CHO cell size increase phase in fed-batch cultures
Source: Appl Microbiol Biotechnol. 2017 Sep 26;101(22):8101–13. doi: 10.1007/s00253-017-8531-y (PMC5656727; doi:10.1007/s00253-017-8531-y)
Supplement: Supplementary file 1 — (PDF 1196 kb) [file 253_2017_8531_MOESM1_ESM.pdf]

Applied Microbiology and Biotechnology

Title

Metabolic characterization of a CHO cell size increase phase in fed-batch cultures

Authors

Xiao Pan [a] (Corresponding author), Ciska Dalm [b], René H. Wijffels [a, c], Dirk E. Martens [a]

[a] Bioprocess Engineering, Wageningen University, PO Box 16, 6700 AA, Wageningen, the Netherlands

[b] Synthon Biopharmaceuticals BV, Upstream Process Development, PO Box 7071, 6503 GN, Nijmegen, the Netherlands

[c] Faculty of Biosciences and Aquaculture, Nord University, N-8049, Bodø, Norway

Correspondence: Xiao Pan;
E-mail address: xiao.pan@wur.nl
Telephone: +31-317482683

ESM_1 Flux distribution and flux variability analysis

The metabolic model is based on an earlier published general model for mammalian cells (Martens and Tramper, 2009). Several modifications were made based on the Kyoto Encyclopedia of Genes and Genomes (KEGG) database, the iCHOv1 model, and the measurements done in this study:

1. To allow for production or uptake of the organic acids, acetic acid, fumaric acid, formic acid, citric acid, isovaleric acid, 2-oxobutanoic acid, pyruvic acid, and succinic acid transport steps over the membrane were included in the model. Furthermore, for isovaleric acid and 2-oxobutanoic acid pathways for threonine, serine and leucine degradation, which were lumped in the original model, were split based on KEGG pathways to allow for the synthesis of these compounds. Thioesterases were assumed to be responsible for the formation of acetic acid from acetyl-coA and isovaleric acid from isovaleric acid-CoA.
2. The fatty acid composition was measured and included in the lipid synthesis reaction.
3. The reaction for the monoclonal antibody product was modified to the known amino acid composition of IgG1.
4. The biomass equation was modified based on the biomass analysis for both the NI and the SI phases in this study and thus different biomass equations were used for these phases. The amount of biomass made in both equations corresponds to the mass of 10^9 cells from the NI phase.

Flux balance analysis

		NI Phase constrains		SI Phase constrains		Flux distribution	
Abbreviation	Reaction	Lower bound	Upper bound	Lower bound	Upper bound	NI Phase	SI Phase
2_7_1_1	Glc[c] + ATP[c] <=> ADP[c] + H[c] + G6P[c]	0	10000	0	10000	4.489	4.069
L_PPP1	G6P[c] + H2O[c] + 2 NADP[c] <=> 2 H[c] + CO2[c] + R5P[c] + 2 NADPH[c]	0	10000	0	10000	2.642	2.983
L_R5P1	R5P[c] <=> X5P[c]	-10000	10000	-10000	10000	1.666	1.932
2_2_1_1a	R5P[c] + X5P[c] <=> S7P[c] + GAP[c]	-10000	10000	-10000	10000	0.833	0.966
2_2_1_2	S7P[c] + GAP[c] <=> E4P[c] + F6P[c]	-10000	10000	-10000	10000	0.833	0.966

2_2_1_1b	X5P[c] + E4P[c] <=> GAP[c] + F6P[c]	-10000	10000	-10000	10000	0.833	0.966
5_3_1_9	G6P[c] <=> F6P[c]	-10000	10000	-10000	10000	1.707	0.956
2_7_1_11	ATP[c] + F6P[c] <=> ADP[c] + H[c] + F16P[c]	0	10000	0	10000	3.373	2.889
3_1_3_11	H2O[c] + F16P[c] <=> F6P[c] + Pi[c]	0	10000	0	10000	0.000	0.000
L_GLC1	F16P[c] <=> 2 GAP[c]	-10000	10000	-10000	10000	3.373	2.889
L_GLC2	ADP[c] + GAP[c] + Pi[c] + NAD[c] <=> ATP[c] + H[c] + 3PG[c] + NADH[c]	0	10000	0	10000	7.504	6.657
L_GLC3	3PG[c] <=> H2O[c] + PEP[c]	-10000	10000	-10000	10000	7.567	6.657
2_7_1_40	ADP[c] + H[c] + PEP[c] <=> ATP[c] + PYR[c]	0	10000	0	10000	7.567	6.657
1_1_1_27	H[c] + NADH[c] + PYR[c] <=> NAD[c] + LAC[c]	-10000	10000	-10000	10000	3.103	-0.201
T_Pyr	PYR[c] <=> PYR[m]	-10000	10000	-10000	10000	1.961	4.902
E012	PYR[m] + CoA[m] + NAD[m] <=> CO2[c] + AcCoA[m] + NADH[m]	0	10000	0	10000	4.823	7.547
3_1_2_1	H2O[c] + AcCoA[m] <=> H[c] + CoA[m] + ACE[m]	0	10000	0	10000	0.051	0.216
T_ACE	ACE[m] <=> ACE[c]	-10000	10000	-10000	10000	0.051	0.216
4_1_3_7	H2O[c] + AcCoA[m] + OXA[m] <=> H[c] + CoA[m] + CIT[m]	0	10000	0	10000	5.275	7.758
L_TCA1	NAD[m] + CIT[m] <=> CO2[c] + NADH[m] + AKG[m]	0	10000	0	10000	3.230	5.394
L_TCA2	CoA[m] + NAD[m] + AKG[m] <=> CO2[c] + NADH[m] + SUCCoA[m]	0	10000	0	10000	2.715	5.059
6_2_1_4	Pi[c] + SUCCoA[m] + GDP[c] <=> CoA[m] + SUC[m] + GTP[c]	0	10000	0	10000	2.695	5.102
1_3_99_1	SUC[m] + FAD[m] <=> FADH2[m] + FUM[m]	-10000	10000	-10000	10000	3.451	5.499
4_2_1_2b	H2O[c] + FUM[m] <=> MAL[m]	-10000	10000	-10000	10000	3.583	5.615
1_1_1_37b	NAD[m] + MAL[m] <=> H[c] + NADH[m] + OXA[m]	-10000	10000	-10000	10000	2.697	3.824
6_4_1_1	ATP[c] + CO2[c] + H2O[c] + PYR[m] <=> ADP[c] + 2 H[c] + Pi[c] + OXA[m]	0	10000	0	10000	2.564	3.754
4_1_1_32a	CO2[c] + PEP[c] + GDP[c] <=> GTP[c] + OXA[c]	-10000	0	-10000	0	0.000	0.000
1_1_1_40a	NADP[c] + MAL[c] <=> CO2[c] + NADPH[c] + PYR[c]	-10000	10000	-10000	10000	-1.681	-1.913
1_1_1_40b	NAD[m] + MAL[m] <=> CO2[c] + PYR[m] + NADH[m]	-10000	10000	-10000	10000	5.238	6.297
T_SUC	SUC[m] <=> SUC[c]	-10000	10000	-10000	10000	-0.490	-0.166

2_6_1_2	PYR[c] + GLU[c] <=> AKG[c] + ALA[c]	-10000	10000	-10000	10000	0.820	0.183
3_5_1_1	H2O[c] + ASN[c] <=> NH4[c] + ASP[c]	0	10000	0	10000	0.849	0.395
3_5_1_2	H2O[c] + GLN[c] <=> NH4[c] + GLU[m]	0	10000	0	10000	0.000	0.000
1_4_1_3a	H2O[c] + NAD[m] + GLU[m] <=> H[c] + NADH[m] + AKG[m] + NH4[c]	-10000	10000	-10000	10000	-0.156	0.426
L_PRO1	H2O[c] + NAD[m] + PRO[c] <=> H[c] + NADH[m] + G5S[m]	-10000	10000	-10000	10000	0.815	2.072
L_G5S1	H2O[c] + G5S[m] + NADP[m] <=> 2 H[c] + GLU[m] + NADPH[m]	-10000	10000	-10000	10000	0.770	2.046
L_CYS1	AKG[m] + NADPH[m] + 2 O2[c] + CYS[c] <=> H[c] + PYR[m] + GLU[m] + NADP[m] + SO4[c]	0	10000	0	10000	0.188	0.102
L_GLYCLE	NAD[m] + GLY[c] + THF[c] <=> CO2[c] + NADH[m] + NH4[c] + METHF[c]	-10000	10000	-10000	10000	-0.084	-0.086
2_1_2_1a	H2O[c] + GLY[c] + METHF[c] <=> THF[c] + SER[c]	-10000	10000	-10000	10000	-0.411	-0.257
4_3_1_17	SER[c] <=> PYR[c] + NH4[c]	0	10000	0	10000	0.000	0.142
L_THR1	CoA[m] + NAD[m] + THR[c] <=> H[c] + AcCoA[m] + NADH[m] + GLY[c]	0	10000	0	10000	0.027	0.020
4_3_1_19	THR[c] <=> NH4[c] + OBU[m]	0	1000	0	1000	0.019	0.025
L_LYSII	H2O[c] + CoA[m] + 3 NAD[m] + 2 AKG[m] + NADPH[m] + LYS[c] <=> 2 H[c] + CO2[c] + 3 NADH[m] + 2 GLU[m] + NADP[m] + GLUTCoA[m]	0	10000	0	10000	0.000	0.001
L_TRYPII	3 H2O[c] + NADPH[c] + CoA[m] + NAD[m] + 3 O2[c] + TRYP[c] <=> H[c] + 2 CO2[c] + NADP[c] + NADH[m] + ALA[c] + NH4[c] + GLUTCoA[m] + FORM[c]	0	10000	0	10000	0.066	0.028
L_GLUCOA	H2O[c] + CoA[m] + NAD[m] + FAD[m] + GLUTCoA[m] <=> CO2[c] + 2 AcCoA[m] + NADH[m] + FADH2[m]	0	10000	0	10000	0.066	0.029
L_MET1	ATP[c] + 2 H2O[c] + THF[c] + MET[c] <=> H[c] + Pi[c] + Ad[c] + HCYS[c] + MTHF[c] + PPi[c]	0	10000	0	10000	0.004	0.010
L_HCYS	SER[c] + HCYS[c] <=> NH4[c] + CYS[c] + OBU[m]	-10000	10000	-10000	10000	0.004	0.010
T_OBU	OBU[m] <=> OBU[c]	-10000	10000	-10000	10000	0.023	0.036
L_OBU	CoA[m] + NAD[m] + OBU[m] <=> CO2[c] + NADH[m] + PROPCoA[m]	-10000	10000	-10000	10000	0.000	0.000
L_ILE1	H2O[c] + 2 CoA[m] + 2 NAD[m] + FAD[m] + AKG[c] + ILE[c] <=> H[c] + CO2[c] + AcCoA[m] + 2 NADH[m] + FADH2[m] + GLU[c] + PROPCoA[m]	0	10000	0	10000	0.081	0.077
L_VAL1	2 H2O[c] + CoA[m] + 3 NAD[m] + FAD[m] + AKG[c] + VAL[c] <=> 2 H[c] + 2 CO2[c] + 3 NADH[m] +	0	10000	0	10000	0.031	0.096

	FADH2[m] + GLU[c] + PROPCoA[m]						
L_PropCoA	CO2[c] + H2O[c] + PROPCoA[m] + ATP[m] <=> 2 H[c] + Pi[c] + SUCCoA[m] + ADP[m]	0	10000	0	10000	0.112	0.173
L_PHE1	H[c] + NADH[m] + O2[c] + PHE[c] <=> H2O[c] + NAD[m] + TYR[c]	-10000	10000	-10000	10000	0.013	0.049
L_TYR1	H2O[c] + AKG[m] + 2 O2[c] + TYR[c] <=> 2 H[c] + CO2[c] + FUM[m] + GLU[m] + AcAc[m]	0	10000	0	10000	0.132	0.116
L_ACAC1	CoA[m] + SUCCoA[m] + AcAc[m] <=> 2 AcCoA[m] + SUC[m]	0	10000	0	10000	0.132	0.130
L_HIS1	2 H[c] + 2 H2O[c] + THF[c] + HIS[c] <=> 2 NH4[c] + GLU[m] + MYLTHF[c]	0	10000	0	10000	0.012	0.011
L_LEU1	CoA[m] + NAD[m] + AKG[m] + LEU[c] <=> CO2[c] + NADH[m] + GLU[m] + IVACoA[m]	0	10000	0	10000	0.057	0.116
L_LEU2	CO2[c] + 2 H2O[c] + FAD[m] + ATP[m] + IVACoA[m] <=> 2 H[c] + Pi[c] + AcCoA[m] + FADH2[m] + ADP[m] + AcAc[m]	0	10000	0	10000	0.000	0.013
Eiva	H2O[c] + IVACoA[m] <=> H[c] + CoA[m] + IVA[m]	0	10000	0	10000	0.057	0.103
T_IVA	IVA[m] <=> IVA[c]	-10000	10000	-10000	10000	0.057	0.103
6_3_1_2	ATP[c] + GLU[c] + NH4[c] <=> ADP[c] + H[c] + Pi[c] + GLN[c]	0	10000	0	10000	0.240	0.358
L_PRO_S	ATP[c] + 2 H[c] + 2 NADH[c] + GLU[c] <=> ADP[c] + H2O[c] + Pi[c] + 2 NAD[c] + PRO[c]	0	10000	0	10000	0.972	2.167
1_14_11_2	AKG[c] + PRO[c] + O2[c] <=> CO2[c] + SUC[m] + HYP[c]	0	10000	0	10000	0.135	0.102
L_SER_S	H2O[c] + 3PG[c] + NAD[c] + GLU[c] <=> H[c] + Pi[c] + NADH[c] + AKG[c] + SER[c]	-10000	10000	-10000	10000	-0.063	0.000
6_3_5_4	ATP[c] + H2O[c] + ASP[c] + GLN[c] <=> H[c] + GLU[c] + ASN[c] + PPi[c] + AMP[c]	0	10000	0	10000	0.000	0.000
3_5_3_1a	H2O[c] + ARG[c] <=> ORN[c] + Urea[c]	0	10000	0	10000	0.000	0.000
T_ORn	ORN[c] <=> ORN[m]	-10000	10000	-10000	10000	0.000	0.000
6_3_4_16	CO2[c] + H2O[c] + NH4[c] + 2 ATP[m] <=> 3 H[c] + Pi[c] + 2 ADP[m] + CaP[m]	0	10000	0	10000	0.045	0.026
2_1_3_3	ORN[m] + CaP[m] <=> H[c] + Pi[c] + Citr[m]	-10000	10000	-10000	10000	0.045	0.026
T_CITR	Citr[m] <=> Citr[c]	-10000	10000	-10000	10000	0.045	0.026
6_3_4_5	ATP[c] + ASP[c] + Citr[c] <=> PPi[c] + AMP[c] + ArgSuc[c]	0	10000	0	10000	0.045	0.026
4_3_2_1	ArgSuc[c] <=> H[c] + ARG[c] + FUM[c]	-10000	10000	-10000	10000	0.045	0.026
4_2_1_2a	H2O[c] + FUM[c] <=> MAL[c]	-10000	10000	-10000	10000	0.118	0.081
2_6_1_13	AKG[m] + ORN[m] <=> GLU[m] + G5S[m]	-10000	10000	-10000	10000	-0.045	-0.026

3_5_4_9b	ATP[c] + THF[c] + FORM[c] <=> ADP[c] + Pi[c] + N10FTHF[c]	-10000	10000	-10000	10000	-0.191	-0.108
3_5_4_9a	H2O[c] + MYLTHF[c] <=> H[c] + N10FTHF[c]	-10000	10000	-10000	10000	0.334	0.193
1_5_1_15	NADH[c] + MYLTHF[c] <=> NAD[c] + METHF[c]	-10000	10000	-10000	10000	-0.322	-0.181
1_5_1_20	H[c] + NADPH[c] + METHF[c] <=> NADP[c] + MTHF[c]	-10000	10000	-10000	10000	-0.004	-0.010
1_5_1_6	H2O[c] + NAD[c] + N10FTHF[c] <=> H[c] + CO2[c] + NADH[c] + THF[c]	0	10000	0	10000	0.000	0.000
T_MAL	MAL[c] <=> MAL[m]	-10000	10000	-10000	10000	4.338	4.234
T_GLU	GLU[c] <=> GLU[m]	-10000	10000	-10000	10000	-1.270	-1.943
1_1_1_37a	NAD[c] + MAL[c] <=> H[c] + NADH[c] + OXA[c]	-10000	10000	-10000	10000	-2.553	-2.512
SLC25A11	AKG[m] + MAL[c] <=> MAL[m] + AKG[c]	-10000	10000	-10000	10000	0.014	0.271
2_6_1_1b	OXA[m] + GLU[m] <=> AKG[m] + ASP[m]	-10000	10000	-10000	10000	-0.014	-0.179
SLC1A3	GLU[c] + ASP[m] <=> ASP[c] + GLU[m]	-10000	10000	-10000	10000	-0.014	-0.179
2_6_1_1a	OXA[c] + GLU[c] <=> AKG[c] + ASP[c]	-10000	10000	-10000	10000	-0.524	-0.179
1_6_1_2	NADPH[c] + NAD[c] <=> NADP[c] + NADH[c]	0	10000	0	10000	0.000	0.000
1_6_1_2b	NAD[m] + NADPH[m] <=> NADH[m] + NADP[m]	0	10000	0	10000	0.582	1.942
L_NADH1	4 H[c] + 3 Pi[c] + NADH[m] + 0.5 O2[c] + 3 ADP[m] <=> 4 H2O[c] + NAD[m] + 3 ATP[m]	0	10000	0	10000	20.318	33.064
L_FAD1	2 H[c] + 2 Pi[c] + FADH2[m] + 0.5 O2[c] + 2 ADP[m] <=> 3 H2O[c] + FAD[m] + 2 ATP[m]	0	10000	0	10000	3.629	5.714
T_ADPATP	ADP[c] + ATP[m] <=> ATP[c] + ADP[m]	-10000	10000	-10000	10000	68.011	110.382
2_7_4_6	ATP[c] + GDP[c] <=> ADP[c] + GTP[c]	-10000	10000	-10000	10000	-2.652	-5.077
2_7_4_3	ATP[c] + AMP[c] <=> 2 ADP[c]	0	10000	0	10000	0.221	0.137
2_7_1_20	ATP[c] + Ad[c] <=> ADP[c] + H[c] + AMP[c]	-10000	10000	-10000	10000	0.004	0.010
3_6_1_3	ATP[c] + H2O[c] <=> ADP[c] + H[c] + Pi[c]	0	10000	0	10000	52.380	97.266
3_6_1_1	H2O[c] + PPi[c] <=> H[c] + 2 Pi[c]	0	10000	0	10000	0.149	0.095
L_ATP1	6 ATP[c] + CO2[c] + H2O[c] + R5P[c] + GTP[c] + 2 ASP[c] + 2 GLN[c] + GLY[c] + 2 N10FTHF[c] <=> 6 ADP[c] + 10 H[c] + 5 Pi[c] + GDP[c] + 2 GLU[c] + 2 THF[c] + PPi[c] + AMP[c] + 2 FUM[c]	0	10000	0	10000	0.043	0.025
L_GTP	8 ATP[c] + CO2[c] + 2 H2O[c] + R5P[c] + NAD[c] + NH4[c] + ASP[c] + 2 GLN[c] + GLY[c] + 2 N10FTHF[c]	0	10000	0	10000	0.029	0.017

	<=> 6 ADP[c] + 11 H[c] + 4 Pi[c] + NADH[c] + GTP[c] + 2 GLU[c] + 2 THF[c] + 2 PPi[c] + 2 AMP[c] + FUM[c]						
L_UTP2	3 ATP[c] + R5P[c] + ASP[c] + GLN[c] + 0.5 O2[c] <=> 2 ADP[c] + 2 H[c] + H2O[c] + 2 Pi[c] + GLU[c] + AMP[c] + UTP[c]	0	10000	0	10000	0.072	0.042
6_3_4_2	ATP[c] + NH4[c] + UTP[c] <=> ADP[c] + 2 H[c] + Pi[c] + CTP[c]	0	10000	0	10000	0.029	0.017
L_RNA1	0.3 ATP[c] + H2O[c] + 0.2 GTP[c] + 0.3 UTP[c] + 0.2 CTP[c] <=> H[c] + 2 Pi[c] + RNA[c]	0	10000	0	10000	0.112	0.085
L_DNA2	0.3 ATP[c] + 0.3 H[c] + NADPH[c] + 0.3 NADH[c] + 0.2 GTP[c] + 0.3 METHF[c] + 0.3 UTP[c] + 0.2 CTP[c] <=> NADP[c] + 2 Pi[c] + 0.3 NAD[c] + 0.3 THF[c] + DNA[c]	0	10000	0	10000	0.031	0.000
L_TC1	ATP[c] + G6P[c] + H2O[c] <=> ADP[c] + H[c] + 2 Pi[c] + TC[c]	0	10000	0	10000	0.139	0.128
T_CIT	CIT[m] <=> CIT[c]	-10000	10000	-10000	10000	2.046	2.363
4_1_3_8	ATP[c] + CIT[c] + CoA[c] <=> ADP[c] + Pi[c] + OXA[c] + AcCoA[c]	0	10000	0	10000	2.029	2.332
L_C14_0	6 ATP[c] + 5 H[c] + H2O[c] + 12 NADPH[c] + 7 AcCoA[c] <=> 6 ADP[c] + 12 NADP[c] + 6 Pi[c] + 7 CoA[c] + FA14_0[c]	0	10000	0	10000	0.003	0.004
L_C16_0	7 ATP[c] + 6 H[c] + H2O[c] + 14 NADPH[c] + 8 AcCoA[c] <=> 7 ADP[c] + 14 NADP[c] + 7 Pi[c] + 8 CoA[c] + FA16_0[c]	0	10000	0	10000	0.146	0.169
L_C16_1	H[c] + NADH[c] + O2[c] + FA16_0[c] <=> 2 H2O[c] + NAD[c] + FA16_1[c]	0	10000	0	10000	0.024	0.028
L_C18_0	ATP[c] + H[c] + 2 NADPH[c] + AcCoA[c] + FA16_0[c] <=> ADP[c] + 2 NADP[c] + Pi[c] + CoA[c] + FA18_0[c]	0	10000	0	10000	0.092	0.107
L_C18_1	H[c] + NADH[c] + O2[c] + FA18_0[c] <=> 2 H2O[c] + NAD[c] + OLE[c]	0	10000	0	10000	0.081	0.094
L_C20_0	ATP[c] + H[c] + 2 NADPH[c] + AcCoA[c] + FA18_0[c] <=> ADP[c] + 2 NADP[c] + Pi[c] + CoA[c] + FA20_0[c]	0	10000	0	10000	0.002	0.003
L_C20_1	H[c] + NADH[c] + O2[c] + FA20_0[c] <=> 2 H2O[c] + NAD[c] + FA20_1[c]	0	10000	0	10000	0.002	0.002
L_PHOA1	3 H[c] + GAP[c] + NADH[c] + 0.044 FA14_0[c] + 0.402 FA16_0[c] + 0.32 FA16_1[c] + 0.115 FA18_0[c] + 1.086 OLE[c] + 0.005 FA20_0[c] + 0.028 FA20_1[c] <=> 2 H2O[c] + NAD[c] + PHOA[c]	0	10000	0	10000	0.075	0.086
L_CHOL1	18 ATP[c] + 7 H[c] + 30 NADPH[c] + 11 O2[c] + 18 AcCoA[c] <=> 18 ADP[c] + 8 CO2[c] + 3 H2O[c] + 30 NADP[c] + 18 Pi[c] + FORM[c] + 18 CoA[c] + CHOL[c]	0	10000	0	10000	0.041	0.047

L_PROT2	4.3 ATP[c] + 3.3 H2O[c] + 0.08 ALA[c] + 0.06 GLU[c] + 0.04 ASN[c] + 0.048 ASP[c] + 0.058 GLN[c] + 0.053 PRO[c] + 0.015 CYS[c] + 0.091 GLY[c] + 0.059 SER[c] + 0.052 THR[c] + 0.069 LYS[c] + 0.008 TRYP[c] + 0.018 MET[c] + 0.043 ILE[c] + 0.061 VAL[c] + 0.034 PHE[c] + 0.025 TYR[c] + 0.02 HIS[c] + 0.081 LEU[c] + 0.035 HYP[c] + 0.049 ARG[c] <=> 4.3 ADP[c] + 4.3 H[c] + 4.3 Pi[c] + Protein[c]	0	10000	0	10000	3.846	2.906
L_BIOMASS1	0.153 RNA[c] + 0.042 DNA[c] + 0.19 TC[c] + 0.102 PHOA[c] + 0.056 CHOL[c] + 5.241 Protein[c] <=> BIOMASS[e]	0	10000	0	0	0.734	0.000
L_BIO_size	0.13 RNA[c] + 0.197 TC[c] + 0.133 PHOA[c] + 0.072 CHOL[c] + 4.47 Protein[c] <=> BIOMASS[e]	0	0	0	10000	0.000	0.650
L_MoAB1	4.3 ATP[c] + 0.012 G6P[c] + 3.3 H2O[c] + 0.0015 NADPH[c] + 0.0015 PEP[c] + 0.067 ALA[c] + 0.0442 GLU[c] + 0.0351 ASN[c] + 0.0413 ASP[c] + 0.0643 GLN[c] + 0.0671 PRO[c] + 0.0248 CYS[c] + 0.07 GLY[c] + 0.0548 SER[c] + 0.0816 THR[c] + 0.0723 LYS[c] + 0.0155 TRYP[c] + 0.0145 MET[c] + 0.0465 ILE[c] + 0.089 VAL[c] + 0.0372 PHE[c] + 0.0424 TYR[c] + 0.0155 HIS[c] + 0.071 LEU[c] + 0.0455 ARG[c] + 0.0075 AcCoA[c] <=> 4.3 ADP[c] + 4.3 H[c] + 0.0015 NADP[c] + 4.3135 Pi[c] + 0.0075 CoA[c] + PROD[c]	0	10000	0	10000	0.064	0.145
MT_H2O	H2O[c] <=> H2O[e]	-10000	10000	-10000	10000	18.181	24.848
MT_Pi	Pi[c] <=> Pi[e]	-10000	10000	-10000	10000	-0.218	-0.171
MT_SO4	SO4[c] <=> SO4[e]	-10000	10000	-10000	10000	0.188	0.102
MT_H	H[c] <=> H[e]	-10000	10000	-10000	10000	2.904	-0.344
MT_O2	O2[c] <=> O2[e]	-10000	10000	-10000	10000	-13.553	-20.720
MT_CO2	CO2[c] <=> CO2[e]	-10000	10000	-10000	10000	15.083	22.335
MT_Glc	Glc[c] <=> GLc[e]	-10000	10000	-10000	10000	-4.489	-4.069
MT_Lac	LAC[c] <=> LAC[e]	-10000	10000	-10000	10000	3.103	-0.201
MT_PYR	PYR[c] <=> PYR[e]	-10000	10000	-10000	10000	0.003	0.002
MT_ACE	ACE[c] <=> ACE[e]	0	10000	0	10000	0.051	0.216
MT_FORM	FORM[c] <=> FORM[e]	-10000	10000	-10000	10000	0.298	0.183
MT_FUM	FUM[c] <=> FUM[e]	-10000	10000	-10000	10000	0.041	0.012
MT_SUC	SUC[c] <=> SUC[e]	-10000	10000	-10000	10000	-0.490	-0.166

MT_CIT	CIT[c] <=> CIT[e]	-10000	10000	-10000	10000	0.016	0.031
MT_OBU	OBU[c] <=> OBU[e]	-10000	10000	-10000	10000	0.023	0.036
MT_IVA	IVA[c] <=> IVA[e]	-10000	10000	-10000	10000	0.057	0.103
MT_NH4	NH4[c] <=> NH4[e]	-10000	10000	-10000	10000	0.380	0.545
MT_LYS	LYS[c] <=> LYS[e]	-10000	10000	-10000	10000	-0.270	-0.212
MT_TRP	TRYP[c] <=> TRYP[e]	-10000	10000	-10000	10000	-0.098	-0.053
MT_ILE	ILE[c] <=> ILE[e]	-10000	10000	-10000	10000	-0.249	-0.208
MT_VAL	VAL[c] <=> VAL[e]	-10000	10000	-10000	10000	-0.271	-0.287
MT_LEU	LEU[c] <=> LEU[e]	-10000	10000	-10000	10000	-0.373	-0.362
MT_PHE	PHE[c] <=> PHE[e]	-10000	10000	-10000	10000	-0.146	-0.154
MT_TYR	TYR[c] <=> TYR[e]	-10000	10000	-10000	10000	-0.218	-0.146
MT_HIS	HIS[c] <=> HIS[e]	-10000	10000	-10000	10000	-0.090	-0.072
MT_THR	THR[c] <=> THR[e]	-10000	10000	-10000	10000	-0.251	-0.208
MT_SER	SER[c] <=> SER[e]	-10000	10000	-10000	10000	-0.709	-0.588
MT_GLY	GLY[c] <=> GLY[e]	-10000	10000	-10000	10000	0.096	0.046
MT_Gln	GLN[c] <=> GLN[e]	-10000	10000	-10000	10000	-0.202	0.054
MT_GLU	GLU[c] <=> GLU[e]	-10000	10000	-10000	10000	-0.069	-0.288
MT_ALA	ALA[c] <=> ALA[e]	-10000	10000	-10000	10000	0.574	-0.032
MT_PRO	PRO[c] <=> PRO[e]	-10000	10000	-10000	10000	-0.186	-0.170
MT_ASN	ASN[c] <=> ASN[e]	-10000	10000	-10000	10000	-1.005	-0.516
MT_ASP	ASP[c] <=> ASP[e]	-10000	10000	-10000	10000	-0.107	-0.245
MT_Cys	CYS[c] <=> CYS[e]	-10000	10000	-10000	10000	-0.243	-0.139
MT_MET	MET[c] <=> MET[e]	-10000	10000	-10000	10000	-0.074	-0.065
MT_ARG	ARG[c] <=> ARG[e]	-10000	10000	-10000	10000	-0.146	-0.123
MT_Urea	Urea[c] <=> Urea[e]	-10000	10000	-10000	10000	0.000	0.000

MT_Prod	PROD[c] <=> PROD[e]	0	10000	0	10000	0.064	0.145
Ex_H2O	H2O[e] <=>	-10000	10000	-10000	10000	18.181	24.848
Ex_Pi	Pi[e] <=>	-10000	10000	-10000	10000	-0.218	-0.171
Ex_SO4	SO4[e] <=>	-10000	10000	-10000	10000	0.188	0.102
Ex_H	H[e] <=>	-10000	10000	-10000	10000	2.904	-0.344
Ex_O2	O2[e] <=>	-10000	10000	-10000	10000	-13.553	-20.720
Ex_CO2	CO2[e] <=>	-10000	10000	-10000	10000	15.083	22.335
Ex_GLc	GLc[e] <=>	-4.623	-4.489	-4.134	-4.069	-4.489	-4.069
Ex_LAC	LAC[e] <=>	3.068	3.103	-0.308	-0.201	3.103	-0.201
Ex_PYR	PYR[e] <=>	-0.020	0.003	-0.041	0.002	0.003	0.002
Ex_ACE	ACE[e] <=>	0.044	0.051	0.179	0.216	0.051	0.216
Ex_FORM	FORM[e] <=>	0.272	0.298	0.161	0.183	0.298	0.183
Ex_FUM	FUM[e] <=>	0.037	0.041	0.010	0.012	0.041	0.012
Ex_SUC	SUC[e] <=>	-0.511	-0.490	-0.184	-0.166	-0.490	-0.166
Ex_CIT	CIT[e] <=>	0.006	0.016	0.024	0.031	0.016	0.031
Ex_OBU	OBU[e] <=>	0.014	0.023	0.029	0.036	0.023	0.036
Ex_IVA	IVA[e] <=>	0.049	0.057	0.091	0.103	0.057	0.103
Ex_NH4	NH4[e] <=>	0.380	0.440	-0.250	1.000	0.380	0.545
Ex_LYS	LYS[e] <=>	-0.270	-0.213	-0.224	-0.212	-0.270	-0.212
Ex_TRYP	TRYP[e] <=>	-0.107	-0.098	-0.059	-0.053	-0.098	-0.053
Ex_ILE	ILE[e] <=>	-0.263	-0.249	-0.216	-0.208	-0.249	-0.208
Ex_VAL	VAL[e] <=>	-0.323	-0.271	-0.302	-0.287	-0.271	-0.287
Ex_LEU	LEU[e] <=>	-0.393	-0.360	-0.394	-0.362	-0.373	-0.362
Ex_PHE	PHE[e] <=>	-0.162	-0.146	-0.159	-0.154	-0.146	-0.154
Ex_TYR	TYR[e] <=>	-0.218	-0.218	-0.159	-0.146	-0.218	-0.146

Ex_HIS	HIS[e] <=>	-0.090	-0.082	-0.079	-0.072	-0.090	-0.072
Ex_THR	THR[e] <=>	-0.269	-0.251	-0.233	-0.208	-0.251	-0.208
Ex_SER	SER[e] <=>	-0.734	-0.709	-0.650	-0.588	-0.709	-0.588
Ex_GLY	GLY[e] <=>	0.096	0.141	0.025	0.046	0.096	0.046
Ex_GLN	GLN[e] <=>	-0.251	-0.174	0.041	0.054	-0.202	0.054
Ex_GLU	GLU[e] <=>	-0.091	-0.069	-0.411	-0.288	-0.069	-0.288
Ex_ALA	ALA[e] <=>	0.459	0.574	-0.073	-0.032	0.574	-0.032
Ex_PRO	PRO[e] <=>	-0.244	-0.186	-0.185	-0.170	-0.186	-0.170
Ex_ASN	ASN[e] <=>	-1.005	-0.941	-0.558	-0.516	-1.005	-0.516
Ex_ASP	ASP[e] <=>	-0.143	-0.107	-0.250	-0.245	-0.107	-0.245
Ex_CYS	CYS[e] <=>	-0.262	-0.243	-0.152	-0.139	-0.243	-0.139
Ex_MET	MET[e] <=>	-0.084	-0.074	-0.069	-0.065	-0.074	-0.065
Ex_ARG	ARG[e] <=>	-0.146	-0.132	-0.131	-0.123	-0.146	-0.123
Ex_Urea	Urea[e] <=>	0.000	0.000	0.000	0.000	0.000	0.000
Ex_BIOMASS	BIOMASS[e] <=>	0.720	0.750	0.530	0.650	0.734	0.650
Ex_PROD	PROD[e] <=>	0.064	0.075	0.136	0.145	0.064	0.145

Flux variability analysis

		Flux variability analysis			
**Abbreviation**	**Reaction**	**NI Min**	**NI Max**	**SI Min**	**SI Max**
2_7_1_1	Glc[c] + ATP[c] <=> ADP[c] + H[c] + G6P[c]	4.489	4.623	4.069	4.134
L_PPP1	G6P[c] + H2O[c] + 2 NADP[c] <=> 2 H[c] + CO2[c] + R5P[c] + 2 NADPH[c]	0.000	13.212	0.000	21.575
L_R5P1	R5P[c] <=> X5P[c]	-0.095	8.713	-0.056	14.327
2_2_1_1a	R5P[c] + X5P[c] <=> S7P[c] + GAP[c]	-0.048	4.356	-0.028	7.164
2_2_1_2	S7P[c] + GAP[c] <=> E4P[c] + F6P[c]	-0.048	4.356	-0.028	7.164
2_2_1_1b	X5P[c] + E4P[c] <=> GAP[c] + F6P[c]	-0.048	4.356	-0.028	7.164
5_3_1_9	G6P[c] <=> F6P[c]	-8.730	4.482	-17.571	4.004
2_7_1_11	ATP[c] + F6P[c] <=> ADP[c] + H[c] + F16P[c]	0.000	75.288	0.000	126.030
3_1_3_11	H2O[c] + F16P[c] <=> F6P[c] + Pi[c]	0.000	70.901	0.000	122.082
L_GLC1	F16P[c] <=> 2 GAP[c]	-0.017	4.387	-3.244	3.948
L_GLC2	ADP[c] + GAP[c] + Pi[c] + NAD[c] <=> ATP[c] + H[c] + 3PG[c] + NADH[c]	4.247	8.651	0.589	7.781
L_GLC3	3PG[c] <=> H2O[c] + PEP[c]	-62.182	8.775	-114.057	8.038
2_7_1_40	ADP[c] + H[c] + PEP[c] <=> ATP[c] + PYR[c]	0.000	10000.000	0.000	10000.000
1_1_1_27	H[c] + NADH[c] + PYR[c] <=> NAD[c] + LAC[c]	3.068	3.103	-0.308	-0.201
T_Pyr	PYR[c] <=> PYR[m]	-22.362	10000.000	-38.054	10000.000
E012	PYR[m] + CoA[m] + NAD[m] <=> CO2[c] + AcCoA[m] + NADH[m]	1.802	6.340	2.017	9.276
3_1_2_1	H2O[c] + AcCoA[m] <=> H[c] + CoA[m] + ACE[m]	0.044	0.051	0.179	0.216
T_ACE	ACE[m] <=> ACE[c]	0.044	0.051	0.179	0.216
4_1_3_7	H2O[c] + AcCoA[m] + OXA[m] <=> H[c] + CoA[m] + CIT[m]	2.420	6.923	2.524	9.729
L_TCA1	NAD[m] + CIT[m] <=> CO2[c] + NADH[m] + AKG[m]	0.385	4.887	0.168	7.373
L_TCA2	CoA[m] + NAD[m] + AKG[m] <=> CO2[c] + NADH[m] + SUCCoA[m]	0.000	4.404	0.000	7.197

6_2_1_4	Pi[c] + SUCCoA[m] + GDP[c] <=> CoA[m] + SUC[m] + GTP[c]	0.000	4.436	0.000	7.222
1_3_99_1	SUC[m] + FAD[m] <=> FADH2[m] + FUM[m]	0.756	5.259	0.415	7.702
4_2_1_2b	H2O[c] + FUM[m] <=> MAL[m]	0.888	5.408	0.531	7.838
1_1_1_37b	NAD[m] + MAL[m] <=> H[c] + NADH[m] + OXA[m]	-132.470	10000.000	-229.809	10000.000
6_4_1_1	ATP[c] + CO2[c] + H2O[c] + PYR[m] <=> ADP[c] + 2 H[c] + Pi[c] + OXA[m]	0.000	70.901	0.000	122.082
4_1_1_32a	CO2[c] + PEP[c] + GDP[c] <=> GTP[c] + OXA[c]	-10000.000	0.000	-10000.000	0.000
1_1_1_40a	NADP[c] + MAL[c] <=> CO2[c] + NADPH[c] + PYR[c]	-22.820	9999.542	-39.096	9999.010
1_1_1_40b	NAD[m] + MAL[m] <=> CO2[c] + PYR[m] + NADH[m]	-9998.406	90.414	-9998.102	151.589
T_SUC	SUC[m] <=> SUC[c]	-0.511	-0.490	-0.184	-0.166
2_6_1_2	PYR[c] + GLU[c] <=> AKG[c] + ALA[c]	0.696	0.820	0.135	0.183
3_5_1_1	H2O[c] + ASN[c] <=> NH4[c] + ASP[c]	0.785	24.419	0.395	41.091
3_5_1_2	H2O[c] + GLN[c] <=> NH4[c] + GLU[m]	0.000	70.901	0.000	122.082
1_4_1_3a	H2O[c] + NAD[m] + GLU[m] <=> H[c] + NADH[m] + AKG[m] + NH4[c]	-70.855	0.066	-121.077	1.012
L_PRO1	H2O[c] + NAD[m] + PRO[c] <=> H[c] + NADH[m] + G5S[m]	0.233	71.159	0.122	122.241
L_G5S1	H2O[c] + G5S[m] + NADP[m] <=> 2 H[c] + GLU[m] + NADPH[m]	0.188	71.099	0.104	122.216
L_CYS1	AKG[m] + NADPH[m] + 2 O2[c] + CYS[c] <=> H[c] + PYR[m] + GLU[m] + NADP[m] + SO4[c]	0.188	0.217	0.102	0.120
L_GLYCLE	NAD[m] + GLY[c] + THF[c] <=> CO2[c] + NADH[m] + NH4[c] + METHF[c]	-0.142	4.298	-0.115	7.113
2_1_2_1a	H2O[c] + GLY[c] + METHF[c] <=> THF[c] + SER[c]	-4.800	-0.370	-7.410	-0.201
4_3_1_17	SER[c] <=> PYR[c] + NH4[c]	0.000	70.901	0.000	122.082
L_THR1	CoA[m] + NAD[m] + THR[c] <=> H[c] + AcCoA[m] + NADH[m] + GLY[c]	0.000	0.064	0.000	0.071
4_3_1_19	THR[c] <=> NH4[c] + OBU[m]	0.000	0.064	0.000	0.071
L_LYSII	H2O[c] + CoA[m] + 3 NAD[m] + 2 AKG[m] + NADPH[m] + LYS[c] <=> 2 H[c] + CO2[c] + 3 NADH[m] + 2 GLU[m] + NADP[m] + GLUTCoA[m]	0.000	0.000	0.001	0.014
L_TRYPII	3 H2O[c] + NADPH[c] + CoA[m] + NAD[m] + 3 O2[c] + TRYP[c] <=> H[c] + 2 CO2[c] + NADP[c] + NADH[m] + ALA[c] + NH4[c] + GLUTCoA[m] + FORM[c]	0.066	0.075	0.028	0.033

L_GLUCOA	H2O[c] + CoA[m] + NAD[m] + FAD[m] + GLUTCoA[m] <=> CO2[c] + 2 AcCoA[m] + NADH[m] + FADH2[m]	0.066	0.075	0.029	0.047
L_MET1	ATP[c] + 2 H2O[c] + THF[c] + MET[c] <=> H[c] + Pi[c] + Ad[c] + HCYS[c] + MTHF[c] + PPi[c]	0.004	0.014	0.010	0.015
L_HCYS	SER[c] + HCYS[c] <=> NH4[c] + CYS[c] + OBU[m]	0.004	0.014	0.010	0.015
T_OBU	OBU[m] <=> OBU[c]	0.014	0.023	0.029	0.036
L_OBU	CoA[m] + NAD[m] + OBU[m] <=> CO2[c] + NADH[m] + PROPCoA[m]	-0.019	0.065	-0.025	0.056
L_ILE1	H2O[c] + 2 CoA[m] + 2 NAD[m] + FAD[m] + AKG[c] + ILE[c] <=> H[c] + CO2[c] + AcCoA[m] + 2 NADH[m] + FADH2[m] + GLU[c] + PROPCoA[m]	0.081	0.094	0.077	0.085
L_VAL1	2 H2O[c] + CoA[m] + 3 NAD[m] + FAD[m] + AKG[c] + VAL[c] <=> 2 H[c] + 2 CO2[c] + 3 NADH[m] + FADH2[m] + GLU[c] + PROPCoA[m]	0.031	0.083	0.096	0.113
L_PropCoA	CO2[c] + H2O[c] + PROPCoA[m] + ATP[m] <=> 2 H[c] + Pi[c] + SUCCoA[m] + ADP[m]	0.093	0.242	0.148	0.254
L_PHE1	H[c] + NADH[m] + O2[c] + PHE[c] <=> H2O[c] + NAD[m] + TYR[c]	0.013	0.029	0.049	0.056
L_TYR1	H2O[c] + AKG[m] + 2 O2[c] + TYR[c] <=> 2 H[c] + CO2[c] + FUM[m] + GLU[m] + AcAc[m]	0.132	0.149	0.116	0.136
L_ACAC1	CoA[m] + SUCCoA[m] + AcAc[m] <=> 2 AcCoA[m] + SUC[m]	0.132	0.177	0.130	0.194
L_HIS1	2 H[c] + 2 H2O[c] + THF[c] + HIS[c] <=> 2 NH4[c] + GLU[m] + MYLTHF[c]	0.004	0.012	0.011	0.019
L_LEU1	CoA[m] + NAD[m] + AKG[m] + LEU[c] <=> CO2[c] + NADH[m] + GLU[m] + IVACoA[m]	0.049	0.077	0.116	0.149
L_LEU2	CO2[c] + 2 H2O[c] + FAD[m] + ATP[m] + IVACoA[m] <=> 2 H[c] + Pi[c] + AcCoA[m] + FADH2[m] + ADP[m] + AcAc[m]	0.000	0.029	0.013	0.058
Eiva	H2O[c] + IVACoA[m] <=> H[c] + CoA[m] + IVA[m]	0.049	0.057	0.091	0.103
T_IVA	IVA[m] <=> IVA[c]	0.049	0.057	0.091	0.103
6_3_1_2	ATP[c] + GLU[c] + NH4[c] <=> ADP[c] + H[c] + Pi[c] + GLN[c]	0.191	71.169	0.345	122.427
L_PRO_S	ATP[c] + 2 H[c] + 2 NADH[c] + GLU[c] <=> ADP[c] + H2O[c] + Pi[c] + 2 NAD[c] + PRO[c]	0.332	71.258	0.202	122.320
1_14_11_2	AKG[c] + PRO[c] + O2[c] <=> CO2[c] + SUC[m] + HYP[c]	0.135	0.135	0.102	0.102
L_SER_S	H2O[c] + 3PG[c] + NAD[c] + GLU[c] <=> H[c] + Pi[c] + NADH[c] + AKG[c] + SER[c]	-0.124	70.833	-0.257	121.838
6_3_5_4	ATP[c] + H2O[c] + ASP[c] + GLN[c] <=> H[c] + GLU[c] + ASN[c] + PPi[c] + AMP[c]	0.000	23.634	0.000	40.694
3_5_3_1a	H2O[c] + ARG[c] <=> ORN[c] + Urea[c]	0.000	0.000	0.000	0.000

T_ORn	ORN[c] <=> ORN[m]	0.000	0.000	0.000	0.000
6_3_4_16	CO2[c] + H2O[c] + NH4[c] + 2 ATP[m] <=> 3 H[c] + Pi[c] + 2 ADP[m] + CaP[m]	0.045	0.059	0.018	0.026
2_1_3_3	ORN[m] + CaP[m] <=> H[c] + Pi[c] + Citr[m]	0.045	0.059	0.018	0.026
T_CITR	Citr[m] <=> Citr[c]	0.045	0.059	0.018	0.026
6_3_4_5	ATP[c] + ASP[c] + Citr[c] <=> PPi[c] + AMP[c] + ArgSuc[c]	0.045	0.059	0.018	0.026
4_3_2_1	ArgSuc[c] <=> H[c] + ARG[c] + FUM[c]	0.045	0.059	0.018	0.026
4_2_1_2a	H2O[c] + FUM[c] <=> MAL[c]	0.118	0.136	0.074	0.084
2_6_1_13	AKG[m] + ORN[m] <=> GLU[m] + G5S[m]	-0.059	-0.045	-0.026	-0.018
3_5_4_9b	ATP[c] + THF[c] + FORM[c] <=> ADP[c] + Pi[c] + N10FTHF[c]	-0.191	-0.156	-0.108	-0.081
3_5_4_9a	H2O[c] + MYLTHF[c] <=> H[c] + N10FTHF[c]	0.299	9.107	0.166	14.549
1_5_1_15	NADH[c] + MYLTHF[c] <=> NAD[c] + METHF[c]	-9.103	-0.287	-14.531	-0.147
1_5_1_20	H[c] + NADPH[c] + METHF[c] <=> NADP[c] + MTHF[c]	-0.014	-0.004	-0.015	-0.010
1_5_1_6	H2O[c] + NAD[c] + N10FTHF[c] <=> H[c] + CO2[c] + NADH[c] + THF[c]	0.000	8.808	0.000	14.384
T_MAL	MAL[c] <=> MAL[m]	-9996.946	29.732	-9996.946	41.935
T_GLU	GLU[c] <=> GLU[m]	-71.422	-0.458	-121.636	0.530
1_1_1_37a	NAD[c] + MAL[c] <=> H[c] + NADH[c] + OXA[c]	-10000.000	136.868	-10000.000	236.841
SLC25A11	AKG[m] + MAL[c] <=> MAL[m] + AKG[c]	-139.227	9999.740	-238.765	10000.000
2_6_1_1b	OXA[m] + GLU[m] <=> AKG[m] + ASP[m]	-139.393	9999.540	-239.538	9999.642
SLC1A3	GLU[c] + ASP[m] <=> ASP[c] + GLU[m]	-139.393	9999.540	-239.538	9999.642
2_6_1_1a	OXA[c] + GLU[c] <=> AKG[c] + ASP[c]	-10000.000	138.897	-10000.000	239.174
1_6_1_2	NADPH[c] + NAD[c] <=> NADP[c] + NADH[c]	0.000	10000.000	0.000	10000.000
1_6_1_2b	NAD[m] + NADPH[m] <=> NADH[m] + NADP[m]	0.000	70.901	0.000	122.082
L_NADH1	4 H[c] + 3 Pi[c] + NADH[m] + 0.5 O2[c] + 3 ADP[m] <=> 4 H2O[c] + NAD[m] + 3 ATP[m]	19.424	27.711	32.059	43.987
L_FAD1	2 H[c] + 2 Pi[c] + FADH2[m] + 0.5 O2[c] + 2 ADP[m] <=> 3 H2O[c] + FAD[m] + 2 ATP[m]	0.934	5.540	0.630	8.005
T_ADPATP	ADP[c] + ATP[m] <=> ATP[c] + ADP[m]	67.089	85.016	109.357	133.235

2_7_4_6	ATP[c] + GDP[c] <=> ADP[c] + GTP[c]	-4.393	10000.000	-7.196	10000.000
2_7_4_3	ATP[c] + AMP[c] <=> 2 ADP[c]	0.221	23.879	0.130	40.835
2_7_1_20	ATP[c] + Ad[c] <=> ADP[c] + H[c] + AMP[c]	0.004	0.014	0.010	0.015
3_6_1_3	ATP[c] + H2O[c] <=> ADP[c] + H[c] + Pi[c]	0.000	70.901	0.000	122.082
3_6_1_1	H2O[c] + PPi[c] <=> H[c] + 2 Pi[c]	0.149	23.807	0.088	40.793
L_ATP1	6 ATP[c] + CO2[c] + H2O[c] + R5P[c] + GTP[c] + 2 ASP[c] + 2 GLN[c] + GLY[c] + 2 N10FTHF[c] <=> 6 ADP[c] + 10 H[c] + 5 Pi[c] + GDP[c] + 2 GLU[c] + 2 THF[c] + PPi[c] + AMP[c] + 2 FUM[c]	0.043	0.043	0.025	0.025
L_GTP	8 ATP[c] + CO2[c] + 2 H2O[c] + R5P[c] + NAD[c] + NH4[c] + ASP[c] + 2 GLN[c] + GLY[c] + 2 N10FTHF[c] <=> 6 ADP[c] + 11 H[c] + 4 Pi[c] + NADH[c] + GTP[c] + 2 GLU[c] + 2 THF[c] + 2 PPi[c] + 2 AMP[c] + FUM[c]	0.029	0.029	0.017	0.017
L_UTP2	3 ATP[c] + R5P[c] + ASP[c] + GLN[c] + 0.5 O2[c] <=> 2 ADP[c] + 2 H[c] + H2O[c] + 2 Pi[c] + GLU[c] + AMP[c] + UTP[c]	0.072	0.072	0.042	0.042
6_3_4_2	ATP[c] + NH4[c] + UTP[c] <=> ADP[c] + 2 H[c] + Pi[c] + CTP[c]	0.029	0.029	0.017	0.017
L_RNA1	0.3 ATP[c] + H2O[c] + 0.2 GTP[c] + 0.3 UTP[c] + 0.2 CTP[c] <=> H[c] + 2 Pi[c] + RNA[c]	0.112	0.112	0.084	0.085
L_DNA2	0.3 ATP[c] + 0.3 H[c] + NADPH[c] + 0.3 NADH[c] + 0.2 GTP[c] + 0.3 METHF[c] + 0.3 UTP[c] + 0.2 CTP[c] <=> NADP[c] + 2 Pi[c] + 0.3 NAD[c] + 0.3 THF[c] + DNA[c]	0.031	0.031	0.000	0.000
L_TC1	ATP[c] + G6P[c] + H2O[c] <=> ADP[c] + H[c] + 2 Pi[c] + TC[c]	0.139	0.139	0.128	0.128
T_CIT	CIT[m] <=> CIT[c]	2.035	2.046	2.356	2.363
4_1_3_8	ATP[c] + CIT[c] + CoA[c] <=> ADP[c] + Pi[c] + OXA[c] + AcCoA[c]	2.029	2.029	2.332	2.332
L_C14_0	6 ATP[c] + 5 H[c] + H2O[c] + 12 NADPH[c] + 7 AcCoA[c] <=> 6 ADP[c] + 12 NADP[c] + 6 Pi[c] + 7 CoA[c] + FA14_0[c]	0.003	0.003	0.004	0.004
L_C16_0	7 ATP[c] + 6 H[c] + H2O[c] + 14 NADPH[c] + 8 AcCoA[c] <=> 7 ADP[c] + 14 NADP[c] + 7 Pi[c] + 8 CoA[c] + FA16_0[c]	0.146	0.146	0.169	0.169
L_C16_1	H[c] + NADH[c] + O2[c] + FA16_0[c] <=> 2 H2O[c] + NAD[c] + FA16_1[c]	0.024	0.024	0.028	0.028
L_C18_0	ATP[c] + H[c] + 2 NADPH[c] + AcCoA[c] + FA16_0[c] <=> ADP[c] + 2 NADP[c] + Pi[c] + CoA[c] + FA18_0[c]	0.092	0.092	0.107	0.107
L_C18_1	H[c] + NADH[c] + O2[c] + FA18_0[c] <=> 2 H2O[c] + NAD[c] + OLE[c]	0.081	0.081	0.094	0.094

L_C20_0	ATP[c] + H[c] + 2 NADPH[c] + AcCoA[c] + FA18_0[c] <=> ADP[c] + 2 NADP[c] + Pi[c] + CoA[c] + FA20_0[c]	0.002	0.002	0.003	0.003
L_C20_1	H[c] + NADH[c] + O2[c] + FA20_0[c] <=> 2 H2O[c] + NAD[c] + FA20_1[c]	0.002	0.002	0.002	0.002
L_PHOA1	3 H[c] + GAP[c] + NADH[c] + 0.044 FA14_0[c] + 0.402 FA16_0[c] + 0.32 FA16_1[c] + 0.115 FA18_0[c] + 1.086 OLE[c] + 0.005 FA20_0[c] + 0.028 FA20_1[c] <=> 2 H2O[c] + NAD[c] + PHOA[c]	0.075	0.075	0.086	0.086
L_CHOL1	18 ATP[c] + 7 H[c] + 30 NADPH[c] + 11 O2[c] + 18 AcCoA[c] <=> 18 ADP[c] + 8 CO2[c] + 3 H2O[c] + 30 NADP[c] + 18 Pi[c] + FORM[c] + 18 CoA[c] + CHOL[c]	0.041	0.041	0.047	0.047
L_PROT2	4.3 ATP[c] + 3.3 H2O[c] + 0.08 ALA[c] + 0.06 GLU[c] + 0.04 ASN[c] + 0.048 ASP[c] + 0.058 GLN[c] + 0.053 PRO[c] + 0.015 CYS[c] + 0.091 GLY[c] + 0.059 SER[c] + 0.052 THR[c] + 0.069 LYS[c] + 0.008 TRYP[c] + 0.018 MET[c] + 0.043 ILE[c] + 0.061 VAL[c] + 0.034 PHE[c] + 0.025 TYR[c] + 0.02 HIS[c] + 0.081 LEU[c] + 0.035 HYP[c] + 0.049 ARG[c] <=> 4.3 ADP[c] + 4.3 H[c] + 4.3 Pi[c] + Protein[c]	3.846	3.846	2.905	2.906
L_BIOMASS1	0.153 RNA[c] + 0.042 DNA[c] + 0.19 TC[c] + 0.102 PHOA[c] + 0.056 CHOL[c] + 5.241 Protein[c] <=> BIOMASS[e]	0.734	0.734	0.000	0.000
L_BIO_size	0.13 RNA[c] + 0.197 TC[c] + 0.133 PHOA[c] + 0.072 CHOL[c] + 4.47 Protein[c] <=> BIOMASS[e]	0.000	0.000	0.650	0.650
L_MoAB1	4.3 ATP[c] + 0.012 G6P[c] + 3.3 H2O[c] + 0.0015 NADPH[c] + 0.0015 PEP[c] + 0.067 ALA[c] + 0.0442 GLU[c] + 0.0351 ASN[c] + 0.0413 ASP[c] + 0.0643 GLN[c] + 0.0671 PRO[c] + 0.0248 CYS[c] + 0.07 GLY[c] + 0.0548 SER[c] + 0.0816 THR[c] + 0.0723 LYS[c] + 0.0155 TRYP[c] + 0.0145 MET[c] + 0.0465 ILE[c] + 0.089 VAL[c] + 0.0372 PHE[c] + 0.0424 TYR[c] + 0.0155 HIS[c] + 0.071 LEU[c] + 0.0455 ARG[c] + 0.0075 AcCoA[c] <=> 4.3 ADP[c] + 4.3 H[c] + 0.0015 NADP[c] + 4.3135 Pi[c] + 0.0075 CoA[c] + PROD[c]	0.064	0.064	0.136	0.145
MT_H2O	H2O[c] <=> H2O[e]	18.181	20.256	24.848	27.104
MT_Pi	Pi[c] <=> Pi[e]	-0.218	-0.218	-0.171	-0.171
MT_SO4	SO4[c] <=> SO4[e]	0.188	0.217	0.102	0.120
MT_H	H[c] <=> H[e]	2.583	2.961	-1.217	-0.327
MT_O2	O2[c] <=> O2[e]	-16.114	-13.553	-23.815	-20.720
MT_CO2	CO2[c] <=> CO2[e]	15.083	17.412	22.335	25.433
MT_Glc	Glc[c] <=> GLc[e]	-4.623	-4.489	-4.134	-4.069
MT_Lac	LAC[c] <=> LAC[e]	3.068	3.103	-0.308	-0.201

MT_PYR	PYR[c] <=> PYR[e]	-0.020	0.003	-0.041	0.002
MT_ACE	ACE[c] <=> ACE[e]	0.044	0.051	0.179	0.216
MT_FORM	FORM[c] <=> FORM[e]	0.272	0.298	0.161	0.183
MT_FUM	FUM[c] <=> FUM[e]	0.037	0.041	0.010	0.012
MT_SUC	SUC[c] <=> SUC[e]	-0.511	-0.490	-0.184	-0.166
MT_CIT	CIT[c] <=> CIT[e]	0.006	0.016	0.024	0.031
MT_OBU	OBU[c] <=> OBU[e]	0.014	0.023	0.029	0.036
MT_IVA	IVA[c] <=> IVA[e]	0.049	0.057	0.091	0.103
MT_NH4	NH4[c] <=> NH4[e]	0.380	0.440	0.545	1.000
MT_LYS	LYS[c] <=> LYS[e]	-0.270	-0.270	-0.224	-0.212
MT_TRP	TRYP[c] <=> TRYP[e]	-0.107	-0.098	-0.059	-0.053
MT_ILE	ILE[c] <=> ILE[e]	-0.263	-0.249	-0.216	-0.208
MT_VAL	VAL[c] <=> VAL[e]	-0.323	-0.271	-0.302	-0.287
MT_LEU	LEU[c] <=> LEU[e]	-0.393	-0.365	-0.394	-0.362
MT_PHE	PHE[c] <=> PHE[e]	-0.162	-0.146	-0.159	-0.154
MT_TYR	TYR[c] <=> TYR[e]	-0.218	-0.218	-0.159	-0.146
MT_HIS	HIS[c] <=> HIS[e]	-0.090	-0.082	-0.079	-0.072
MT_THR	THR[c] <=> THR[e]	-0.269	-0.251	-0.233	-0.208
MT_SER	SER[c] <=> SER[e]	-0.734	-0.709	-0.650	-0.588
MT_GLY	GLY[c] <=> GLY[e]	0.096	0.141	0.025	0.046
MT_Gln	GLN[c] <=> GLN[e]	-0.251	-0.174	0.041	0.054
MT_GLU	GLU[c] <=> GLU[e]	-0.091	-0.069	-0.411	-0.288
MT_ALA	ALA[c] <=> ALA[e]	0.459	0.574	-0.073	-0.032
MT_PRO	PRO[c] <=> PRO[e]	-0.244	-0.186	-0.185	-0.170
MT_ASN	ASN[c] <=> ASN[e]	-1.005	-0.941	-0.558	-0.516

MT_ASP	ASP[c] <=> ASP[e]	-0.143	-0.107	-0.250	-0.245
MT_Cys	CYS[c] <=> CYS[e]	-0.262	-0.243	-0.152	-0.139
MT_MET	MET[c] <=> MET[e]	-0.084	-0.074	-0.069	-0.065
MT_ARG	ARG[c] <=> ARG[e]	-0.146	-0.132	-0.131	-0.123
MT_Urea	Urea[c] <=> Urea[e]	0.000	0.000	0.000	0.000
MT_Prod	PROD[c] <=> PROD[e]	0.064	0.064	0.136	0.145
Ex_H2O	H2O[e] <=>	18.181	20.256	24.848	27.104
Ex_Pi	Pi[e] <=>	-0.218	-0.218	-0.171	-0.171
Ex_SO4	SO4[e] <=>	0.188	0.217	0.102	0.120
Ex_H	H[e] <=>	2.583	2.961	-1.217	-0.327
Ex_O2	O2[e] <=>	-16.114	-13.553	-23.815	-20.720
Ex_CO2	CO2[e] <=>	15.083	17.412	22.335	25.433
Ex_GLc	GLc[e] <=>	-4.623	-4.489	-4.134	-4.069
Ex_LAC	LAC[e] <=>	3.068	3.103	-0.308	-0.201
Ex_PYR	PYR[e] <=>	-0.020	0.003	-0.041	0.002
Ex_ACE	ACE[e] <=>	0.044	0.051	0.179	0.216
Ex_FORM	FORM[e] <=>	0.272	0.298	0.161	0.183
Ex_FUM	FUM[e] <=>	0.037	0.041	0.010	0.012
Ex_SUC	SUC[e] <=>	-0.511	-0.490	-0.184	-0.166
Ex_CIT	CIT[e] <=>	0.006	0.016	0.024	0.031
Ex_OBU	OBU[e] <=>	0.014	0.023	0.029	0.036
Ex_IVA	IVA[e] <=>	0.049	0.057	0.091	0.103
Ex_NH4	NH4[e] <=>	0.380	0.440	0.545	1.000
Ex_LYS	LYS[e] <=>	-0.270	-0.270	-0.224	-0.212
Ex_TRYP	TRYP[e] <=>	-0.107	-0.098	-0.059	-0.053

Ex_ILE	ILE[e] <=>	-0.263	-0.249	-0.216	-0.208
Ex_VAL	VAL[e] <=>	-0.323	-0.271	-0.302	-0.287
Ex_LEU	LEU[e] <=>	-0.393	-0.365	-0.394	-0.362
Ex_PHE	PHE[e] <=>	-0.162	-0.146	-0.159	-0.154
Ex_TYR	TYR[e] <=>	-0.218	-0.218	-0.159	-0.146
Ex_HIS	HIS[e] <=>	-0.090	-0.082	-0.079	-0.072
Ex_THR	THR[e] <=>	-0.269	-0.251	-0.233	-0.208
Ex_SER	SER[e] <=>	-0.734	-0.709	-0.650	-0.588
Ex_GLY	GLY[e] <=>	0.096	0.141	0.025	0.046
Ex_GLN	GLN[e] <=>	-0.251	-0.174	0.041	0.054
Ex_GLU	GLU[e] <=>	-0.091	-0.069	-0.411	-0.288
Ex_ALA	ALA[e] <=>	0.459	0.574	-0.073	-0.032
Ex_PRO	PRO[e] <=>	-0.244	-0.186	-0.185	-0.170
Ex_ASN	ASN[e] <=>	-1.005	-0.941	-0.558	-0.516
Ex_ASP	ASP[e] <=>	-0.143	-0.107	-0.250	-0.245
Ex_CYS	CYS[e] <=>	-0.262	-0.243	-0.152	-0.139
Ex_MET	MET[e] <=>	-0.084	-0.074	-0.069	-0.065
Ex_ARG	ARG[e] <=>	-0.146	-0.132	-0.131	-0.123
Ex_Urea	Urea[e] <=>	0.000	0.000	0.000	0.000
Ex_BIOMASS	BIOMASS[e] <=>	0.734	0.734	0.650	0.650
Ex_PROD	PROD[e] <=>	0.064	0.064	0.136	0.145

ESM_2 Extracellular concentrations

	amino acids																					
	alanine	arginine	aspartic acid	asparagine	cysteine	cystine	glutamic acid	glutamine	glycine	histidine	hydroxyproline	isoleucine	leucine	lysine	methionine	phenylalanine	proline	serine	threonine	tryptophan	tyrosine	valine
Sample ID \ units	mM	mM	mM	mM	mM	mM	mM	mM	mM	mM	mM	mM	mM	mM	mM	mM	mM	mM	mM	mM	mM	mM
LOD	0.04	0.01	0.16	0.03	0.02	0.01	0.03	0.03	0.13	0.09	0.22	0.04	0.03	0.01	0.06	0.09	0.19	0.03	0.03	0.13	0.05	0.04
LOQ	0.12	0.04	0.53	0.10	0.05	0.04	0.11	0.11	0.44	0.31	0.74	0.13	0.10	0.04	0.22	0.30	0.62	0.09	0.10	0.43	0.17	0.12
ActiFeed-A Replicate 1	20.45	27.56	50.56	73.53	< LOD	< LOD	85.40	< LOD	< LOD	13.79	< LOD	36.97	60.18	44.09	12.11	36.77	28.12	93.47	48.40	< LOD	< LOD	61.10
ActiFeed-A Replicate 2	19.97	26.18	53.18	77.34	< LOD	< LOD	89.94	< LOD	< LOD	13.48	< LOD	35.98	55.18	43.03	11.55	35.84	26.43	98.39	46.61	< LOD	< LOD	59.10
ActiFeed-A Replicate 3	19.23	27.37	52.06	75.58	< LOD	< LOD	87.93	< LOD	< LOD	13.93	< LOD	37.83	60.26	44.11	11.88	37.18	27.64	95.98	47.97	< LOD	< LOD	61.03
ActiFeed-B Replicate 1	< LOD	< LOD	<LOD	<LOD	< LOD	162.43	<LOD	< LOD	< LOD	< LOD	< LOD	< LOD	< LOD	< LOD	< LOD	< LOD	< LOD	<LOD	< LOD	89.21	248.62	< LOD
ActiFeed-B Replicate 2	< LOD	< LOD	<LOD	<LOD	< LOD	160.66	<LOD	< LOD	< LOD	< LOD	< LOD	< LOD	< LOD	< LOD	< LOD	< LOD	< LOD	<LOD	< LOD	89.50	246.42	< LOD
ActiFeed-B Replicate 3	< LOD	< LOD	<LOD	<LOD	< LOD	156.54	<LOD	< LOD	< LOD	< LOD	< LOD	< LOD	< LOD	< LOD	< LOD	< LOD	< LOD	<LOD	< LOD	84.87	239.63	< LOD
Reactor 1 00	0.20	1.50	2.07	6.08	< LOD	0.29	3.05	3.283	1.00	1.07	0.427	3.46	5.20	2.66	1.42	1.63	7.08	7.10	3.11	1.02	1.16	3.89
Reactor 1 02	0.92	1.15	2.49	4.27	< LOD	0.14	3.95	1.307	1.52	0.95	0.459	2.94	4.42	2.21	1.25	1.38	6.50	6.01	2.96	0.93	0.69	3.31
Reactor 1 03 Before feeding	2.12	0.73	2.52	1.24	< LOD	0.09	4.16	0.223	1.62	0.78	0.401	2.44	3.67	1.57	1.00	1.10	6.00	4.15	2.39	0.78	0.50	2.73
Reactor 1 04 Before feeding	5.74	1.27	4.02	0.40	< LOD	0.04	7.05	0.042	2.01	0.96	0.388	2.74	4.39	2.37	1.15	1.97	5.93	5.11	3.24	0.58	0.28	4.04
Reactor 1 05 Before feeding	6.86	1.76	5.41	0.24	< LOD	0.26	10.19	0.040	2.36	1.11	0.347	2.96	4.76	2.92	1.23	2.69	6.16	5.41	3.97	0.65	0.55	4.66
Reactor 1 06 Before feeding	7.06	2.04	5.95	0.15	< LOD	0.52	12.07	0.161	2.65	1.25	0.337	3.19	4.84	3.29	1.23	3.29	5.96	5.04	4.69	0.72	0.88	5.52
Reactor 1 07 Before feeding	7.67	2.43	6.62	0.12	< LOD	0.82	13.84	0.503	2.64	1.38	0.295	3.41	4.82	3.85	1.37	3.83	6.19	5.64	5.06	0.74	0.95	6.05
Reactor 1 08 Before feeding	8.53	2.98	7.43	0.16	< LOD	0.92	15.06	0.843	3.18	1.57	0.325	3.82	5.60	4.59	1.53	4.55	6.30	6.64	5.71	0.85	1.15	7.37
Reactor 1 09 Before feeding	9.11	3.50	8.65	0.19	< LOD	1.25	17.95	0.931	3.12	1.84	0.281	4.55	6.66	5.31	1.70	5.33	6.52	8.07	6.76	1.00	1.28	8.56
Reactor 1 10 Before feeding	8.59	4.40	10.04	0.24	< LOD	1.45	22.92	1.117	3.86	2.06	0.293	5.39	7.82	6.57	2.02	5.98	7.09	9.73	7.45	1.17	1.48	9.76
Reactor 2 00	NA	1.37	2.08	5.01	< LOD	0.31	3.00	5.17	0.82	0.97	0.37	3.38	5.33	2.54	1.45	1.50	6.46	6.74	3.16	0.93	0.65	3.78
Reactor 2 02	1.33	1.19	2.30	3.19	< LOD	0.21	3.17	1.77	1.24	0.90	0.33	2.82	4.45	2.30	1.34	1.32	6.30	5.11	3.25	0.86	0.53	3.26
Reactor 2 03 Before feeding	2.39	0.73	2.21	1.10	< LOD	0.12	3.50	0.36	1.53	0.72	0.33	2.25	3.55	1.60	1.07	1.01	5.70	3.71	2.76	0.76	0.38	2.66
Reactor 2 03 After feeding	3.32	1.74	3.86	2.86	< LOD	0.19	6.78	0.15	1.27	1.06	0.31	3.38	5.50	3.05	1.45	2.15	5.81	6.57	4.60	0.69	0.46	4.50
Reactor 2 04 Before feeding	5.24	1.48	3.61	0.36	< LOD	0.12	6.51	0.00	2.03	0.90	0.31	2.66	4.36	2.75	1.26	1.87	6.03	4.54	3.56	0.56	0.29	3.77
Reactor 2 04 After feeding	5.86	2.39	5.33	2.12	< LOD	0.72	9.64	0.00	1.74	1.34	0.32	3.74	6.26	4.03	1.67	3.09	6.71	7.09	5.09	0.80	0.77	5.68
Reactor 2 05 Before feeding	6.08	1.54	4.68	0.19	< LOD	0.37	9.21	0.04	1.95	1.07	0.29	3.02	4.79	2.66	1.36	2.55	6.00	4.66	4.20	0.63	0.51	4.81
Reactor 2 06 Before feeding	5.81	2.00	5.92	0.12	< LOD	0.60	12.06	0.189	2.36	1.12	0.313	2.94	4.27	3.26	1.23	2.99	6.00	5.16	4.04	0.70	0.58	5.08
Reactor 2 06 After feeding	6.14	2.95	7.00	1.74	< LOD	1.21	14.44	0.30	2.06	1.40	0.23	3.98	6.29	4.75	1.72	3.95	6.24	7.34	6.23	0.81	0.88	6.96
Reactor 2 07 Before feeding	6.52	2.27	5.96	0.09	< LOD	0.89	12.31	0.71	2.46	1.24	0.35	3.46	5.03	3.69	1.48	3.66	5.86	5.12	5.46	0.68	0.81	6.17
Reactor 2 07 After feeding	6.44	3.14	7.60	1.76	< LOD	1.42	16.15	0.64	1.98	1.59	0.22	4.17	6.75	4.95	1.96	4.59	6.48	7.47	7.06	0.82	1.01	7.86
Reactor 2 08 Before feeding	6.98	2.59	6.91	0.09	< LOD	1.07	13.59	0.94	2.53	1.44	< LOD	3.84	5.57	4.20	1.70	4.21	5.59	5.09	6.44	0.70	0.83	7.08
Reactor 2 08 After feeding	7.29	3.75	8.36	1.56	< LOD	1.70	18.72	0.91	2.12	1.88	< LOD	4.71	7.57	5.99	2.03	5.40	6.85	7.92	8.00	0.89	1.14	9.01
Reactor 2 09 Before feeding	7.48	2.77	7.94	0.17	< LOD	1.11	14.61	1.39	2.53	1.64	< LOD	4.34	6.61	4.37	1.84	5.06	5.83	7.27	7.32	0.70	1.31	8.35
Reactor 2 10 Before feeding	7.56	4.17	10.55	0.24	< LOD	1.35	22.11	1.147	3.46	2.07	0.279	5.38	7.77	6.23	1.93	6.13	6.73	10.21	7.71	1.07	1.15	9.80
Reactor 3 00	NA	1.27	1.83	4.67	< LOD	0.29	2.75	4.71	0.77	0.99	0.33	3.13	4.93	2.40	1.31	1.45	6.07	6.31	2.79	0.92	0.73	3.52
Reactor 3 02	1.33	1.05	2.23	3.06	< LOD	0.18	3.12	1.65	1.14	0.93	0.33	2.77	4.36	1.98	1.28	1.33	6.14	4.95	3.14	0.89	0.60	3.19
Reactor 3 03 Before feeding	2.44	0.72	2.12	0.97	< LOD	0.12	3.40	0.30	1.60	0.73	0.32	2.19	3.46	1.57	1.01	1.02	5.21	3.59	3.34	0.76	0.39	2.54
Reactor 3 03 After feeding	3.34	1.50	3.85	2.55	< LOD	0.18	6.29	0.13	1.17	1.08	0.26	3.24	5.18	2.67	1.44	2.11	6.27	6.16	3.90	0.75	0.47	4.27
Reactor 3 04 Before feeding	5.01	1.31	3.59	0.30	< LOD	0.11	6.40	0.00	1.93	0.95	0.35	2.66	4.29	2.42	1.26	1.87	5.38	4.41	3.50	0.64	0.31	3.75
Reactor 3 04 After feeding	5.42	1.71	5.19	2.04	< LOD	0.51	9.54	0.00	1.45	1.20	0.27	3.57	5.76	2.90	1.61	2.82	6.49	7.04	4.90	0.70	0.70	5.39
Reactor 3 05 Before feeding	5.54	1.52	4.56	0.16	< LOD	0.36	8.84	0.04	2.18	0.97	0.30	2.80	4.43	2.66	1.24	2.37	5.77	4.38	3.84	0.54	0.50	4.47
Reactor 3 06 Before feeding	6.41	1.85	5.87	0.10	< LOD	0.56	12.02	0.192	2.30	1.14	0.326	3.02	4.64	3.16	1.18	3.13	6.10	5.01	4.19	0.64	0.72	5.59
Reactor 3 06 After feeding	6.13	2.38	6.74	1.56	< LOD	1.02	10.94	0.28	1.85	1.51	0.25	4.00	6.25	3.80	1.69	3.88	6.25	6.76	5.84	0.75	0.69	6.91
Reactor 3 07 Before feeding	6.58	2.20	5.71	0.07	< LOD	0.81	10.51	0.67	2.37	1.15	0.25	3.19	4.65	3.58	1.38	3.46	5.51	4.99	5.12	0.58	0.63	5.84
Reactor 3 07 After feeding	7.34	3.22	7.84	1.44	< LOD	1.30	13.50	0.58	2.30	1.49	0.24	4.28	6.58	5.11	1.82	4.50	6.04	6.99	7.06	0.75	1.07	7.69
Reactor 3 08 Before feeding	7.23	2.96	6.12	0.08	< LOD	1.21	13.67	0.98	2.90	1.28	0.28	3.51	4.92	4.82	1.54	4.06	5.09	5.19	5.97	0.59	0.84	6.59
Reactor 3 08 After feeding	7.09	3.21	8.00	1.45	< LOD	1.55	14.44	0.92	2.03	1.69	0.32	4.65	6.79	5.05	1.97	4.94	5.71	7.81	7.42	0.84	1.53	8.33
Reactor 3 09 Before feeding	7.46	3.39	7.27	0.10	< LOD	1.48	15.81	1.23	2.87	1.54	0.26	4.18	5.87	5.31	1.79	4.68	5.65	6.22	6.88	0.75	1.13	7.77
Reactor 3 10 Before feeding	7.46	3.90	9.68	0.20	< LOD	1.36	20.93	1.127	3.27	2.04	0.285	5.06	7.29	5.75	1.80	5.91	6.51	9.39	7.53	1.01	1.40	9.46

	saccharides									organic acids													her organic compoun		
	fucose	galactose	glucose	glucose-1-phosphate	lactose	mannose	raffinose	sucrose	xylose	acetic acid	butyric&2-hydroxybutyric acids	3-hydroxybutyric acid	citric acid	formic acid	fumaric acid	isovaleric acid	lactic acid	maleic acid	propionic acid	pyruvic acid	succinic acid	acetone	ethanol	pyroglutamic acid
Sample ID \ units	mM	mM	mM	mM	mM	mM	mM	mM	mM	mM	mM	mM	mM	mM	mM	mM	mM	mM	mM	mM	mM	mM	mM	mM
LOD	0.31	0.44	0.31	0.26	0.18	0.16	0.14	0.13	0.31	0.01	0.03	0.04	0.10	0.03	0.01	0.02	0.03	0.02	0.07	0.02	0.01	0.005	0.04	0.22
LOQ	1.04	1.46	1.04	0.87	0.60	0.53	0.48	0.43	1.03	0.03	0.09	0.12	0.32	0.10	0.05	0.07	0.10	0.05	0.22	0.07	0.04	0.017	0.15	0.67
ActiFeed-A Replicate 1	< LOD	< LOD	513.05	< LOD	< LOD	< LOD	< LOD	< LOD	< LOD	< LOD	< LOD	0.37	< LOD	0.04	< LOD	< LOD	< LOD	< LOD	< LOD	1.83	0.50	< LOD	0.45	< LOD
ActiFeed-A Replicate 2	< LOD	< LOD	499.75	< LOD	< LOD	< LOD	< LOD	< LOD	< LOD	< LOD	< LOD	0.37	< LOD	0.05	< LOD	< LOD	< LOD	< LOD	< LOD	1.16	0.29	< LOD	0.47	< LOD
ActiFeed-A Replicate 3	< LOD	< LOD	517.18	< LOD	< LOD	< LOD	< LOD	< LOD	< LOD	< LOD	< LOD	0.38	< LOD	0.04	< LOD	< LOD	< LOD	< LOD	< LOD	2.25	0.24	< LOD	0.52	< LOD
ActiFeed-B Replicate 1	< LOD	< LOD	< LOD	< LOD	< LOD	< LOD	< LOD	< LOD	< LOD	0.25	< LOD	< LOD	< LOD	< LOD	< LOD	< LOD	< LOD	< LOD	< LOD	< LOD	< LOD	< LOD	1.23	< LOD
ActiFeed-B Replicate 2	< LOD	< LOD	< LOD	< LOD	< LOD	< LOD	< LOD	< LOD	< LOD	0.26	< LOD	< LOD	< LOD	0.03	< LOD	< LOD	< LOD	< LOD	< LOD	< LOD	< LOD	< LOD	1.26	< LOD
ActiFeed-B Replicate 3	< LOD	< LOD	< LOD	< LOD	< LOD	< LOD	< LOD	< LOD	< LOD	0.25	< LOD	< LOD	< LOD	< LOD	< LOD	< LOD	< LOD	< LOD	< LOD	< LOD	< LOD	< LOD	1.25	< LOD
Reactor 1 00	< LOD	< LOD	34.26	< LOD	< LOD	< LOD	< LOD	< LOD	< LOD	0.17	< LOD	< LOD	1.03	0.14	< LOD	< LOD	1.17	< LOD	< LOD	0.61	11.28	0.05	0.81	0.372
Reactor 1 02	< LOD	< LOD	22.52	< LOD	< LOD	< LOD	< LOD	< LOD	< LOD	0.22	0.10	0.05	0.95	0.93	0.02	0.06	12.19	< LOD	< LOD	0.85	10.41	0.05	0.88	0.767
Reactor 1 03 Before feeding	< LOD	< LOD	7.92	< LOD	< LOD	< LOD	< LOD	< LOD	< LOD	0.29	0.19	0.11	0.85	1.63	0.12	0.18	23.17	< LOD	< LOD	0.55	9.14	0.09	0.92	0.768
Reactor 1 04 Before feeding	< LOD	< LOD	28.36	< LOD	< LOD	< LOD	< LOD	< LOD	< LOD	0.51	0.26	0.14	0.95	2.70	0.30	0.38	26.46	< LOD	< LOD	0.60	6.51	0.07	1.34	0.869
Reactor 1 05 Before feeding	< LOD	< LOD	31.40	< LOD	< LOD	< LOD	< LOD	< LOD	< LOD	1.12	0.37	0.12	1.01	4.23	0.42	0.98	22.38	< LOD	< LOD	0.24	4.73	0.09	0.77	0.710
Reactor 1 06 Before feeding	< LOD	< LOD	25.70	< LOD	< LOD	< LOD	< LOD	< LOD	< LOD	2.30	0.63	0.12	1.17	5.63	0.49	1.73	20.01	< LOD	< LOD	0.37	3.48	NA	1.48	0.532
Reactor 1 07 Before feeding	< LOD	< LOD	17.44	< LOD	< LOD	< LOD	< LOD	< LOD	< LOD	3.69	0.79	0.10	1.32	6.15	0.51	2.16	18.08	< LOD	< LOD	0.36	2.33	0.11	0.81	0.386
Reactor 1 08 Before feeding	< LOD	< LOD	20.00	< LOD	< LOD	< LOD	< LOD	< LOD	< LOD	6.11	1.14	0.11	1.58	6.75	0.52	2.60	14.22	< LOD	< LOD	0.19	1.59	0.13	1.32	0.286
Reactor 1 09 Before feeding	< LOD	< LOD	21.00	< LOD	< LOD	< LOD	< LOD	< LOD	< LOD	9.36	1.33	0.12	1.74	7.06	0.43	2.84	12.75	< LOD	< LOD	0.15	1.38	0.09	1.57	0.142
Reactor 1 10 Before feeding	< LOD	< LOD	13.96	< LOD	< LOD	< LOD	< LOD	< LOD	< LOD	14.57	1.41	0.10	1.78	7.09	0.41	2.85	11.76	< LOD	< LOD	< LOD	1.03	0.08	1.45	0.148
Reactor 2 00	< LOD	< LOD	32.32	< LOD	< LOD	< LOD	< LOD	< LOD	< LOD	0.15	< LOD	< LOD	1.02	0.12	< LOD	0.05	0.96	< LOD	< LOD	0.73	11.13	0.054	1.61	0.25
Reactor 2 02	< LOD	< LOD	21.72	< LOD	< LOD	< LOD	< LOD	< LOD	< LOD	0.20	0.10	0.06	0.95	0.87	0.03	0.05	12.34	< LOD	< LOD	0.65	10.41	0.029	0.81	0.63
Reactor 2 03 Before feeding	< LOD	< LOD	7.98	< LOD	< LOD	< LOD	< LOD	< LOD	< LOD	0.26	0.13	0.09	0.84	1.51	0.10	0.14	23.38	< LOD	< LOD	0.78	8.74	0.059	1.28	0.61
Reactor 2 03 After feeding	< LOD	< LOD	40.96	< LOD	< LOD	< LOD	< LOD	< LOD	< LOD	0.29	0.14	0.10	0.90	1.56	0.10	0.16	22.77	< LOD	< LOD	0.66	7.89	0.032	3.39	0.58
Reactor 2 04 Before feeding	< LOD	< LOD	31.40	< LOD	< LOD	< LOD	< LOD	< LOD	< LOD	0.46	0.19	0.15	0.92	2.64	0.25	0.38	25.16	< LOD	< LOD	0.64	6.65	0.038	0.84	0.70
Reactor 2 04 After feeding	< LOD	< LOD	45.94	< LOD	< LOD	< LOD	< LOD	< LOD	< LOD	0.63	0.18	0.14	0.97	3.06	0.30	0.53	24.13	< LOD	< LOD	0.48	6.10	0.065	1.13	0.72
Reactor 2 05 Before feeding	< LOD	< LOD	34.73	< LOD	< LOD	< LOD	< LOD	< LOD	< LOD	0.97	0.28	0.13	1.02	4.15	0.37	1.03	19.22	< LOD	< LOD	0.86	4.85	0.035	1.32	0.64
Reactor 2 06 Before feeding	< LOD	< LOD	27.79	< LOD	< LOD	< LOD	< LOD	< LOD	< LOD	1.74	0.54	0.08	1.08	5.04	0.42	1.58	14.63	< LOD	< LOD	0.32	3.18	NA	1.23	0.453
Reactor 2 06 After feeding	< LOD	< LOD	40.01	< LOD	< LOD	< LOD	< LOD	< LOD	< LOD	2.07	0.44	0.10	1.10	5.17	0.43	1.67	15.67	< LOD	< LOD	0.78	3.02	NA	1.16	0.63
Reactor 2 07 Before feeding	< LOD	< LOD	20.67	< LOD	< LOD	< LOD	< LOD	< LOD	< LOD	3.27	0.64	0.08	1.31	6.11	0.51	2.17	15.06	< LOD	< LOD	0.39	2.35	0.075	1.57	0.58
Reactor 2 07 After feeding	< LOD	< LOD	39.83	< LOD	< LOD	< LOD	< LOD	< LOD	< LOD	3.96	0.67	0.13	1.36	5.89	0.48	2.19	12.87	< LOD	< LOD	0.39	2.02	0.084	2.04	0.58
Reactor 2 08 Before feeding	< LOD	< LOD	18.15	< LOD	< LOD	< LOD	< LOD	< LOD	< LOD	5.81	0.83	0.06	1.57	6.61	0.50	2.61	12.80	< LOD	< LOD	NA	1.55	0.080	0.61	0.43
Reactor 2 08 After feeding	< LOD	< LOD	42.78	< LOD	< LOD	< LOD	< LOD	< LOD	< LOD	6.06	0.87	0.10	1.52	6.28	0.47	2.46	13.20	< LOD	< LOD	NA	1.38	0.048	1.14	0.46
Reactor 2 09 Before feeding	< LOD	< LOD	20.65	< LOD	< LOD	< LOD	< LOD	< LOD	< LOD	8.90	1.08	0.07	1.71	6.79	0.46	2.76	11.42	< LOD	< LOD	NA	1.12	0.092	1.52	NA
Reactor 2 10 Before feeding	< LOD	< LOD	12.54	< LOD	< LOD	< LOD	< LOD	< LOD	< LOD	15.00	1.54	0.09	1.84	7.21	0.43	2.91	11.24	< LOD	< LOD	< LOD	0.98	NA	0.45	0.172
Reactor 3 00	< LOD	< LOD	30.29	< LOD	< LOD	< LOD	< LOD	< LOD	< LOD	0.14	< LOD	< LOD	0.94	0.11	< LOD	0.04	0.79	< LOD	< LOD	0.68	10.31	0.048	1.53	0.26
Reactor 3 02	< LOD	< LOD	21.20	< LOD	< LOD	< LOD	< LOD	< LOD	< LOD	0.19	0.11	0.04	0.90	0.88	0.04	0.07	12.56	< LOD	< LOD	0.60	10.28	0.030	1.48	0.64
Reactor 3 03 Before feeding	< LOD	< LOD	7.03	< LOD	< LOD	< LOD	< LOD	< LOD	< LOD	0.26	0.14	0.10	0.78	1.52	0.11	0.14	23.62	< LOD	< LOD	0.78	8.69	0.064	1.39	0.62
Reactor 3 03 After feeding	< LOD	< LOD	40.82	< LOD	< LOD	< LOD	< LOD	< LOD	< LOD	0.30	0.12	0.11	0.89	1.60	0.14	0.17	23.67	< LOD	< LOD	0.85	7.71	0.023	3.12	0.52
Reactor 3 04 Before feeding	< LOD	< LOD	31.82	< LOD	< LOD	< LOD	< LOD	< LOD	< LOD	0.51	0.20	0.12	0.94	2.70	0.29	0.41	25.51	< LOD	< LOD	0.59	6.54	0.048	1.17	0.66
Reactor 3 04 After feeding	< LOD	< LOD	43.47	< LOD	< LOD	< LOD	< LOD	< LOD	< LOD	0.66	0.21	0.11	0.94	2.96	0.32	0.53	22.61	< LOD	< LOD	0.49	5.60	0.070	2.64	0.75
Reactor 3 05 Before feeding	< LOD	< LOD	32.45	< LOD	< LOD	< LOD	< LOD	< LOD	< LOD	1.01	0.28	0.14	0.97	3.99	0.41	1.05	18.81	< LOD	< LOD	0.90	4.45	0.034	2.26	0.76
Reactor 3 06 Before feeding	< LOD	< LOD	27.80	< LOD	< LOD	< LOD	< LOD	< LOD	< LOD	1.90	0.65	0.10	1.17	5.28	0.49	1.73	17.96	< LOD	< LOD	0.22	3.10	0.09	1.67	0.587
Reactor 3 06 After feeding	< LOD	< LOD	38.66	< LOD	< LOD	< LOD	< LOD	< LOD	< LOD	2.15	0.48	0.09	1.10	5.13	0.50	1.71	16.99	< LOD	< LOD	0.42	2.83	0.067	1.59	0.61
Reactor 3 07 Before feeding	< LOD	< LOD	16.94	< LOD	< LOD	< LOD	< LOD	< LOD	< LOD	3.32	0.65	0.08	1.23	5.88	0.52	2.18	17.28	< LOD	< LOD	0.38	2.13	0.067	0.92	0.63
Reactor 3 07 After feeding	< LOD	< LOD	37.67	< LOD	< LOD	< LOD	< LOD	< LOD	< LOD	4.32	0.63	0.11	1.29	5.92	0.47	2.13	16.63	< LOD	< LOD	0.24	2.08	0.069	1.85	0.45
Reactor 3 08 Before feeding	< LOD	< LOD	14.76	< LOD	< LOD	< LOD	< LOD	< LOD	< LOD	5.79	0.93	0.07	1.42	6.39	0.51	2.62	13.61	< LOD	< LOD	NA	1.47	0.082	1.09	0.51
Reactor 3 08 After feeding	< LOD	< LOD	39.51	< LOD	< LOD	< LOD	< LOD	< LOD	< LOD	6.10	0.94	0.11	1.41	6.07	0.48	2.54	13.51	< LOD	< LOD	NA	1.32	0.042	0.98	0.50
Reactor 3 09 Before feeding	< LOD	< LOD	18.71	< LOD	< LOD	< LOD	< LOD	< LOD	< LOD	9.43	1.14	0.05	1.58	6.66	0.48	2.90	10.41	< LOD	< LOD	NA	1.12	0.095	1.73	NA
Reactor 3 10 Before feeding	< LOD	< LOD	11.92	< LOD	< LOD	< LOD	< LOD	< LOD	< LOD	15.20	1.60	0.11	1.74	7.14	0.45	3.08	8.66	< LOD	< LOD	< LOD	0.91	0.10	1.25	0.184

red is below limit of detection

orange is below limit of quantification

blue is not applicable (NA)

"-" is not determined

ESM_3 Supplementary figures

Fig. S1 Specific production (positive values) and consumption rates (negative values) (mmol×10^{-9} cells×day^{-1}) of organic acids during the NI and the SI phase. Error bars show standard deviation of triplicate fed-batch cultures

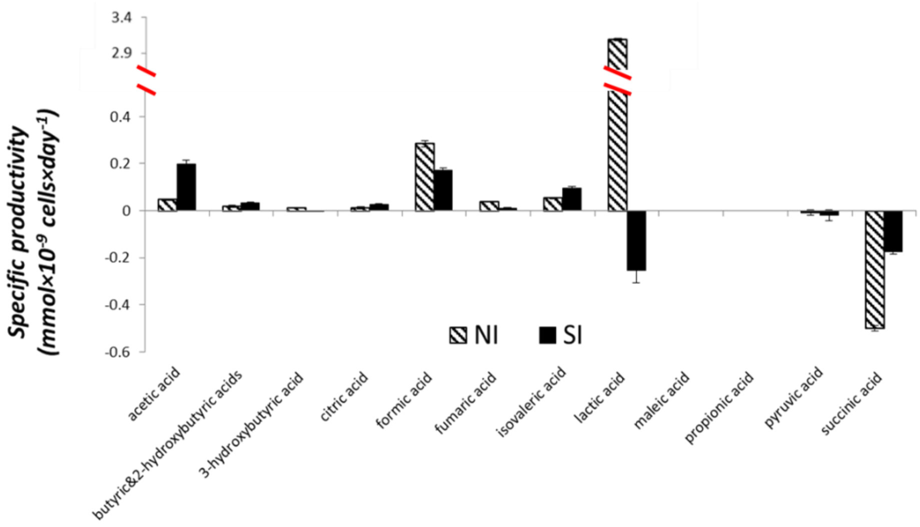

Table S1 Cellular protein composition (g/kg of dry weight)

Protein hydrolysate	Composition (g/kg)	
**Aspartic acid + Asparagine**	**64.89**	±0.59
**Threonine**	**34.78**	±0.49
**Serine**	**34.83**	±0.11
**Glutamic acid + Glutamine**	**96.39**	±0.52
**Proline**	**34.12**	±0.40
**Glycine**	**38.35**	±0.55
**Alanine**	**40.15**	±0.28
**Valine**	**40.03**	±0.44
**Isoleucine**	**31.78**	±0.57
**Leucine**	**59.53**	±0.58
**Tyrosine**	**25.55**	±0.04
**Phenylalanine**	**31.75**	±0.01
**Lysine**	**57.14**	±2.18
**Histidine**	**17.05**	±0.25
**Arginine**	**47.93**	±1.95
**Cysteine**	**10.35**	±0.92
**Methionine**	**14.97**	±0.17
**Tryptophan**	**9.64**	±0.25
**Sum**	**689.19**	

All amino acids were measured by Ansynth Service B.V. on HPLC-UV/FLU after protein hydrolysis

± Deviation from the average value of the duplicate data points on day 4 and day 7

Table S2 Cellular fatty acids composition (w/w %)

Fatty acids	Percentage (w/w)	
**C8:0**	**-**	-
**C10:0**	**-**	-
**C12:0**	**0.1%**	±0.1%
**C14:0**	**1.9%**	±0.4%
**C15:0**	**-**	-
**C16:0**	**18.9%**	±0.8%
**C16:1**	**14.9%**	±0.8%
**C16:2**	**-**	-
**C16:3**	**0.1%**	±0.0%
**C16:4**	**-**	-
**C18:0**	**6.0%**	±0.6%
**C18:1**	**56.2%**	±1.6%
**C18:2**	**-**	-
**C18:3**	**-**	-
**C18:4**	**-**	-
**C19:0**	**-**	-
**C20:0**	**0.3%**	±0.0%
**C20:1**	**1.6%**	±0.1%
**C20:2-n6**	**-**	-
**C20:3-n3**	**-**	-
**C20:4-n6**	**-**	-
**C20:5-n3**	**-**	-
**C22:0**	**-**	-
**C22:1**	**-**	-
**C24:0**	**-**	-

± Standard deviation of six measurements of cells generated from three different culture days (day 4, 7, and 10) with two replicates per culture day
